# A Systematic Review and Meta-Analysis of Cancer Patients Affected by a Novel Coronavirus

**DOI:** 10.1093/jncics/pkaa102

**Published:** 2021-02-24

**Authors:** Bhanu Prasad Venkatesulu, Viveksandeep Thoguluva Chandrasekar, Prashanth Girdhar, Pragati Advani, Amrish Sharma, Thiraviyam Elumalai, Cheng En Hsieh, Hagar I Elghazawy, Vivek Verma, Sunil Krishnan

**Affiliations:** 1 Department of Internal Medicine, Henry Ford Hospital, Detroit, MI, USA; 2 Department of Gastroenterology and Hepatology, University of Kansas Medical Center, Kansas City, KS, USA; 3 Department of Radiation Oncology, All India Institute of Medical Sciences, New Delhi, India; 4 Radiation Epidemiology Branch, Division of Cancer Epidemiology and Genetics, National Institutes of Health, National Cancer Institute, Bethesda, MD, USA; 5 Department of Experimental Radiation Oncology, The University of Texas MD Anderson Cancer Center, Houston, TX, USA; 6 Department of Clinical Oncology, The Christie NHS Foundation Trust, Manchester, UK; 7 Department of Radiation Oncology, Institute for Radiological Research, Chang Gung Memorial Hospital at Linkou and Chang Gung University, Taoyuan City, Taiwan; 8 Graduate School of Biomedical Sciences, The University of Texas Health Science Center at Houston and The University of Texas MD Anderson Cancer Center, Houston, TX, USA; 9 Department of Clinical Oncology, Faculty of Medicine, Ain Shams University, Abbaseya, Cairo, Egypt; 10 Department of Radiation Oncology, Allegheny General Hospital, Pittsburgh, PA, USA; 11 Department of Radiation Oncology, Mayo Clinic Florida, Jacksonville, FL, USA; 12 Department of Radiation Oncology, Loyola University, Maywood, IL

## Abstract

**Background:**

Cancer patients with coronavirus disease 2019 (COVID-19) have been reported to have double the case fatality rate of the general population.

**Methods:**

A systematic search of PubMed, Embase, and Cochrane Central was done for studies on cancer patients with COVID-19. Pooled proportions were calculated for categorical variables. Odds ratio (OR) and forest plots (random-effects model) were constructed for both primary and secondary outcomes.

**Results:**

This systematic review of 38 studies and meta-analysis of 181 323 patients from 26 studies included 23 736 cancer patients. Our meta-analysis shows that cancer patients with COVID-19 have a higher likelihood of death (n = 165 980, OR = 2.54, 95% confidence interval [CI] = 1.47 to 4.42), which was largely driven by mortality among patients in China. Cancer patients were more likely to be intubated. Among cancer subtypes, the mortality was highest in hematological malignancies (n = 878, OR = 2.39, 95% CI = 1.17 to 4.87) followed by lung cancer (n = 646, OR = 1.83, 95% CI = 1.00 to 3.37). There was no association between receipt of a particular type of oncologic therapy and mortality. Our study showed that cancer patients affected by COVID-19 are a decade older than the normal population and have a higher proportion of comorbidities. There was insufficient data to assess the association of COVID-19–directed therapy and survival outcomes in cancer patients.

**Conclusion:**

Cancer patients with COVID-19 disease are at increased risk of mortality and morbidity. A more nuanced understanding of the interaction between cancer-directed therapies and COVID-19–directed therapies is needed. This will require uniform prospective recording of data, possibly in multi-institutional registry databases.

Severe acute respiratory syndrome–related coronavirus 2 (SARS-CoV-2) is a novel beta-coronavirus and the causative agent of coronavirus disease 2019 (COVID-19) (1). COVID-19 has caused an unprecedented global health pandemic, with more than 20 million cases and 0.76 million deaths reported worldwide (at the time of writing) (2). Worldwide data suggest that there are around 18 million new cancer patients every year, with around 43 million patients living with a cancer diagnosis within the past 5 years (3,4). A systematic review showed a pooled prevalence of cancer patients with COVID-19 to be around 2.0% (5). However, cancer patients have been reported to have double the case fatality rates as compared with the general population (6). The majority of cancer patients tend to be older, have multiple preexisting comorbidities, and are immunosuppressed from numerous causes (7). Moreover, owing to oncologic interventions and follow-up thereof, time spent in the hospital as well as interaction with health-care providers may further increase the proclivity to develop infections. For instance, radiotherapy requires multiple visits to the hospital because of its fractionated nature of the treatment and has been known to deplete circulating and resident T-lymphocyte populations (8). Because the main pathophysiologic driver of mortality in COVID-19 is the cytokine storm and macrophage activation, immunotherapy agents might augment the heightened immune activation seen in severe COVID-19 disease (9,10). Lastly, many chemotherapy and targeted therapies require high-dose steroid premedication or therapy and need hospital visits for infusion, both of which predispose to infections.

The COVID-19 pandemic has caused a conundrum of problems specific to cancer patients such as increasing need for intensive care unit (ICU) admissions and ventilatory support; redeployment of resources resulting in delayed cancer care; suspension of clinical trials limiting availability of lifesaving therapies; delay in diagnostic and screening programs; modification of standardized protocols that might compromise cancer control; and reduced willingness among cancer patients to visit hospitals owing to the fear of infection (11,12). The majority of published reports on cancer patients with COVID-19 have been single institutional retrospective studies with selective reporting of outcomes. There remains a multitude of unanswered questions regarding the actual impact of COVID-19 on cancer patients such as differences in survival outcomes in patients with active cancer and cancer survivors, the impact of various oncologic therapies, and difference in outcomes in subtypes of cancer, along with the safety and interaction of COVID-19–directed therapy with cancer-directed therapy. We performed this systematic review and meta-analysis to interrogate and summarize the lessons learned from the clinical reports on various malignancies that have reported mortality outcomes in cancer patients affected by COVID-19.

## Methods

This systematic review was performed according to the Preferred Reporting Items for Systematic Reviews and Meta-Analyses recommendations (13). The complete search protocol is provided in Supplementary Methods (available online). Institutional review board approval was not required for this study because no patient identifiers were disclosed. The systematic review has been registered in the PROSPERO database (CRD42020186671).

### Data Sources

A systematic electronic search was performed in PubMed and MEDLINE, Embase, Cochrane Central, Google Scholar, and MedRxiv databases to identify studies reporting outcomes on cancer patients with COVID-19 from December 1, 2019, to May 23, 2020. The medical subject heading terms used for the search have been provided in the Supplementary Methods (available online). An independent review of the abstracts and full paper articles was performed by 2 reviewers (BV and VC). The duplicates were removed and the titles of articles were then evaluated. Abstracts found to be relevant to the topic of interest were short-listed. Full-length papers of the short-listed articles were assessed for the eligibility criteria. The articles that fulfilled the inclusion criteria were short-listed for final systematic review. The included study references were cross-searched for additional studies. ClinicalTrials.gov, World Health Organization International Clinical Trials Registry Platform, and Cochrane COVID registry were assessed for completed and ongoing clinical trials related to cancer patients with COVID-19. The articles were reviewed independently by 2 authors (BV and VC), and any disagreement was resolved by consensus with a third author (SK). Reasons for excluding studies were documented.

### Study Selection

The inclusion criteria were as follows: studies reporting mortality outcomes in cancer patients with SARS-CoV-2 infection; all types of studies (including randomized controlled trials, prospective, retrospective, and case series) comprising more than 10 patients; and patients aged 18 years and older.

Exclusion criteria were studies that did not report mortality outcomes in cancer patients with SARS-CoV-2 infection and preclinical studies, epidemiological studies, autopsy series, incidence and prevalence studies, or news reports.

### Data Extraction and Quality Assessment

The data was extracted by 2 authors independently into predefined forms. The following data was extracted from the studies: first author, mean age, study design, number of patients, sex, comorbidities, COVID-19–directed treatment, cancer subtypes, different treatments received, number of cancer patients and cancer survivors with corresponding mortality outcomes, ICU admissions, do not resuscitate or do not intubate numbers, need for mechanical ventilation, and progression to severe disease. Data for both cancer and noncancer patients (for available studies) were extracted separately.

### Statistical Analysis

Percentages for categorical variables and medians with interquartile range (IQR) for continuous variables were presented. Pooled rates with 95% confidence intervals (CI) were calculated for individual arms. Odds ratios (OR) comparing cancer patients with noncancer control patients were reported with 95% confidence intervals, and a *P* value less than 0.05 was considered statistically significant. The random-effects model described by DerSimonian and Laird was used for analysis (14). Corresponding forest plots were constructed for both primary and secondary outcomes. Study heterogeneity was assessed using the inconsistency index (*I^2^* statistic) with values of 0%-30%, 31%-60%, 61%-75%, and 76%-100% indicating low, moderate, substantial, and considerable heterogeneity, respectively. All analyses were performed using statistical software Open Meta analyst (CEBM, Brown University, Providence, RI) and Review Manager Version 5.3 (Nordic Cochrane Centre, Copenhagen, Denmark). Subgroup analyses were performed for the following, when data was available: subtypes of cancer, type of cancer-directed therapy, patients with active cancer vs cancer survivors, and mortality outcome based on geographic location.

## Results

### Study Search and Study Characteristics

**Figure 1.**
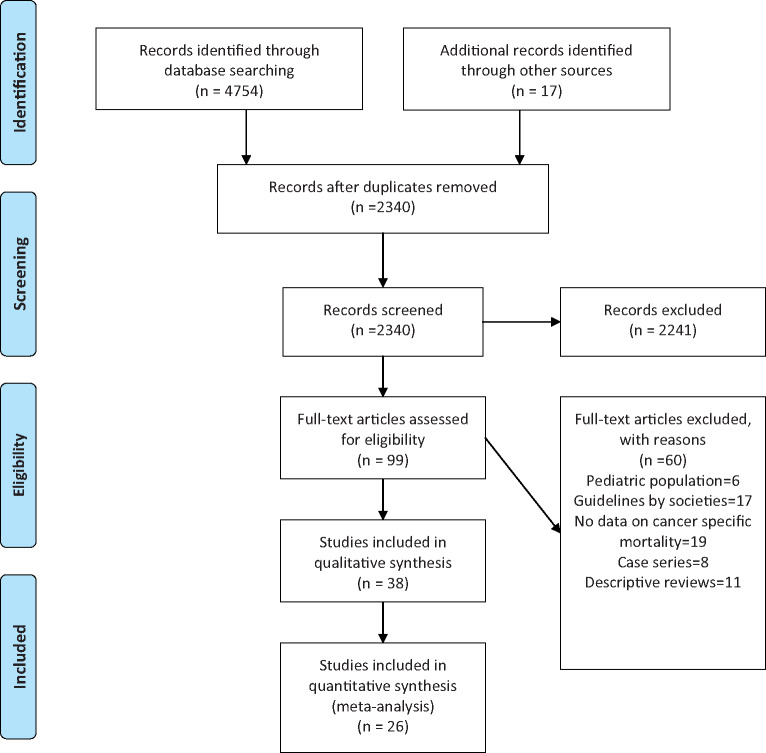
Preferred Reporting Items for Systematic Reviews and Meta-Analyses flow diagram of the selection of studies to be included in the systematic review and meta-analysis.

The literature search resulted in 4754 articles of which 99 articles underwent full review, and 38 were included in the final analysis (Figure 1). Among the studies included for systematic review, 26 were retrospective studies (Supplementary Table 1, available online), and 12 are ongoing clinical trials on cancer patients with no reported outcomes at the time of conducting this meta-analysis (Supplementary Table 2, available online). The data from 26 studies were included in the meta-analysis; 17 studies had multiple cancer types, and 9 studies pertained to a single cancer type. Ten studies were performed in China, 6 in the United States, 3 in the United Kingdom, 3 in Italy, and 2 each in Spain and France. Table 1 summarizes the studies included in the meta-analysis.

### Patient Characteristics

There were 23 736 cancer patients (mean age = 65.1 [8.02] years) vs 157 587 noncancer patients (50.3 [11.9] years). The proportion of males was 52.6% (n = 1413) vs 52.9% (n = 9067), respectively. The proportion of comorbidities was higher in the cancer arm with the prevalence of smoking being 31.1%; hypertension 46.6%; diabetes mellitus 20.4%; cardiac disease 34.9%; cerebrovascular disease 9.1%; chronic liver disease 9.2 %; chronic kidney disease 10.8%; and chronic lung disease 14.7% compared with the noncancer arm with prevalence of smoking being 7.3%; hypertension 24.2%; diabetes mellitus 5.4%; cardiac disease 7.3%; cerebrovascular disease 3.9%; chronic liver disease 6.5%; and chronic kidney disease 4.1%. The demographics are provided in Supplementary Table 3 (available online).

### Primary Outcomes Reported in the Studies

Twenty-six studies provided data on cancer patient mortality. The pooled all-cause in-hospital mortality rate was 19.2% (95% CI = 14.9% to 23.5%) (n = 23 736) with a median follow-up duration of 45 days (range = 21-104 days). Comparing the mortality between the cancer and noncancer patients, 10 studies (n = 165 980) provided such data, with a pooled rate of 16.6% (95% CI = 10.4% to 22.8%) and 5.4% (95% CI = 4.1% to 6.7%), respectively (OR = 2.54, 95% CI = 1.47 to 4.42, *I^2^* = 92%; *P* < .001). Further subgroup analysis performed on studies with sample size over 100 confirmed the aforementioned findings (OR = 2.17, 95% CI = 1.16 to 4.07, *I^2^* = 95%; *P* = .02).

In subgroup analysis stratifying based on geographical location, cancer patients in China had higher mortality than noncancer patients (OR = 6.62, 95% CI = 2.68 to 16.34, *I^2^* = 59%; *P* = .001), but this trend was not observed in the United States (OR = 1.56, 95% CI = 0.62 to 3.96, *I^2^* = 95%; *P* = .35) or Europe (OR = 1.69, 95% CI = 0.81 to 3.52, *I^2^* = 89%; *P* = .16). The forest plots for geographical distribution is shown in Supplementary Figure 1 (available online).

Seventeen studies reported ICU admission rates in cancer patients, with a pooled ICU admission rate of 12.6% (95% CI = 8.9% to 16.3%; n = 1834). When evaluating the 3 studies (n = 11 587), which also provided information on the noncancer population, the pooled ICU admission rates in the respective cohorts were 12.6% (95% CI = 4.7% to 20.5%) and 7.1% (95% CI = 5.1% to 9.1%), with no statistically significant difference between the 2 groups (OR = 2.18, 95% CI = 0.78 to 6.04, *I^2^* = 85%; *P* = .13).

**Figure 2:**
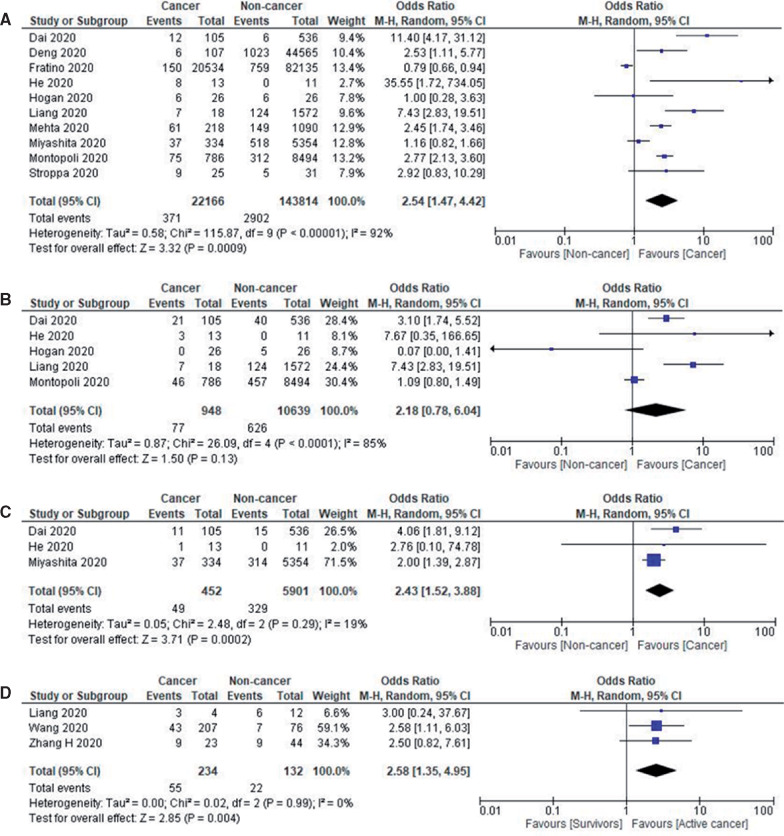
Prognosis of patients with COVID-19. **A)** Forest plot of pooled in-hospital all-cause mortality rates between cancer patients and noncancer patients. **B)** Forest plot of intensive care unit admission rates between cancer patients and noncancer patients. **C)** Forest plot of intubation rates between cancer patients and noncancer patients. **D)** Forest plot of severe disease between active cancer patients and cancer survivors. Odds ratio calculated using the Mantel-Haenszel random-effects model and *P* value from the z test to examine whether the pooled estimate of effect is statistically significant. CI = confidence interval; M-H = Mantel-Haenszel Test.

Fifteen studies reported the need for mechanical ventilation in cancer patients, with a pooled intubation rate of 10.9% (95% CI = 6.8% to 15.0%; n = 1813). When examining the 3 studies (n = 6353), which also provided information on noncancer cases, the pooled rates were 10.8% (95% CI = 7.9% to 13.7%) and 4.9% (95% CI = 2.0% to 7.8%), respectively (OR = 2.43, 95% CI = 1.43 to 3.88, *I^2^* = 19%; *P* < .001). Figure 2 shows the forest plots of prognosis of patients with COVID-19.

### Cancer Subtype-Specific Outcomes

The most common type of cancer reported among COVID-19 patients was hematological malignancies with a reported pooled proportion of 34.3% (95% CI = 7.4% to 61.3%; n = 2316). This was followed by breast cancers at 29.2% (95% CI = 6.1% to 51.40%; n = 1945), lung cancers 23.7% (95% CI = 2.0% to 45.3%; n = 2051), gastrointestinal malignancies 15.2% (95% CI = 11.7% to 18.7%; n = 2643), prostate cancers 11.1% (95% CI = 5.7% to 16.6%; n = 2039), gynecological cancers 9.6% (95% CI = 5.7% to 13.5%; n = 1077), head and neck cancers 3.7% (95% CI = 2.4% to 5.0%; n = 883), brain tumors 3.0% (95% CI = 0.8% to 5.3%; n = 465), and other cancers 2.63% (95% CI = 0.7% to 5.19%; n = 2033). Hematological malignancies had the highest pooled all-cause in-hospital mortality rate of 33.1% (95% CI = 16.1% to 50.1%; n = 266) followed by lung cancer at 28.0% (95% CI = 18.8% to 37.1%; n = 161), gastrointestinal malignancies at 19.8% (95% CI = 6.3% to 33.3%; n = 99), and breast cancer at 10.9% (95% CI = 3.5% to 18.3%; n = 193). The numbers reported in the other malignancies were insufficient to perform a subset analysis.

**Figure 3.**
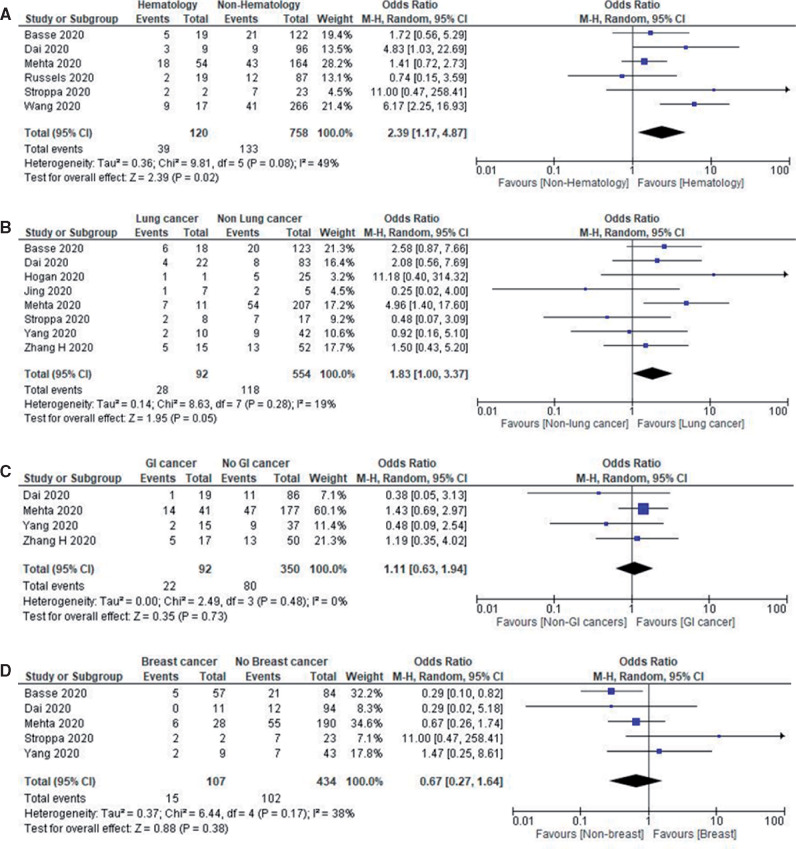
Mortality outcomes in cancer subtypes with COVID-19. **A)** Forest plot of pooled in-hospital all-cause mortality rates between hematological cancer patients and nonhematological cancer patients. **B)** Forest plot of pooled in-hospital all-cause mortality rates between lung cancer patients and nonlung cancer patients. **C)** Forest plot of pooled in-hospital all-cause mortality rates between gastrointestinal cancer patients and nongastrointestinal cancer patients. **D)** Forest plot of in-hospital all-cause mortality rates between breast cancer patients and nonbreast cancer patients. Odds ratio calculated using the Mantel-Haenszel random-effects model and *P* value from the z test to examine whether the pooled estimate of effect is statistically significant. CI = confidence interval; M-H = Mantel-Haenszel Test.

We performed an additional subgroup analysis for mortality by stratifying hematological malignancies vs other malignancies, lung vs other malignancies, gastrointestinal malignancies vs other malignancies, and breast vs other malignancies. Hematological malignancies had the highest odds ratio of death (OR = 2.39, 95% CI = 1.17 to 4.87, *I^2^* = 49%; *P* = .02; n = 878) followed by lung cancer (OR = 1.83, 95% CI = 1.00 to 3.37, *I^2^* = 19%; *P* = .05; n = 646), which were both statistically significant; the remainder were not. Figure 3 shows the forest plots of mortality outcomes in cancer subtypes with COVID-19.

### Treatment-Related Outcomes

**Figure 4.**
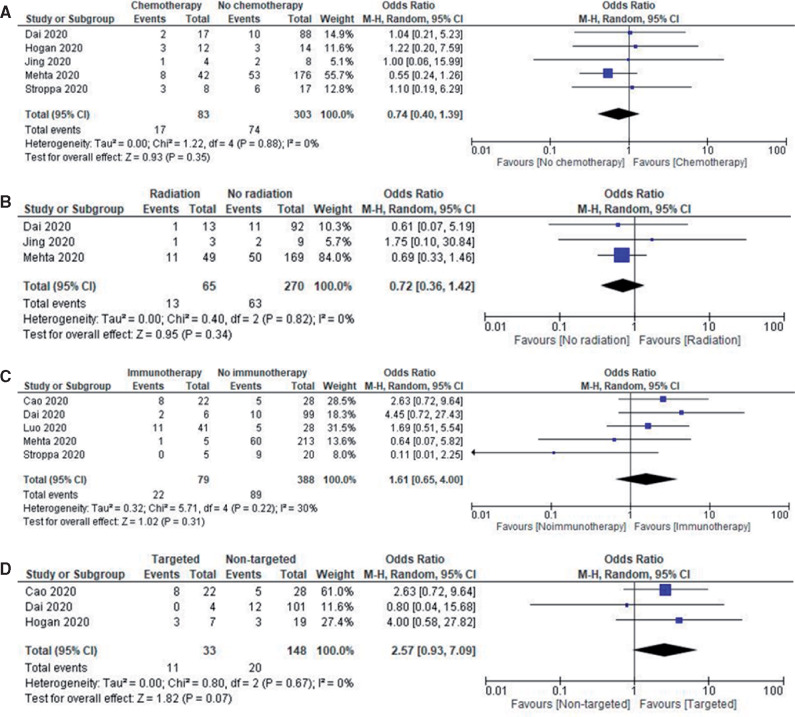
Mortality outcomes with different types of cancer-directed treatment with COVID-19. **A)** Forest plot of pooled in-hospital all-cause mortality rates between patients receiving chemotherapy vs other modalities. **B)** Forest plot of pooled in-hospital all-cause mortality rates between patients receiving radiotherapy vs other modalities. **C)** Forest plot of pooled in-hospital all-cause mortality rates between patients receiving immunotherapy vs other modalities. **D)** Forest plot of pooled-in hospital all-cause mortality rates between patients receiving targeted therapies vs other modalities. Odds ratio calculated using the Mantel-Haenszel random-effects model and *P* value from the z test to examine whether the pooled estimate of effect is statistically significant. CI = confidence interval; M-H = Mantel-Haenszel Test.

The most common treatment modality reported in cancer patients affected with COVID-19 was chemotherapy (pooled rate of 30.3%, 95% CI = 22.3% to 37.8%; n = 1166) followed by hormonal therapy (17.4%, 95% CI = 6.9% to 27.9%; n = 332), targeted therapy 15.4% (95% CI = 9.5% to 21.2%; n = 837), radiotherapy 13.8% (95% CI = 7.0% to 20.7%; n = 790), immunotherapy 9.1% (95% CI = 5.2% to 12.9%; n = 1345), and surgery 7.3% (95% CI = 5.2% to 9.4%; n = 776). Subgroup analysis of treatment modalities showed no statistically significant differences in mortality associated with radiotherapy (OR = 0.72, 95% CI = 0.36 to 1.42, *I^2^* = 0%; *P* = .34; n = 335), chemotherapy (OR = 0.74, 95% CI = 0.40 to 1.39, *I^2^* = 0%; *P* = .35; n = 386), immunotherapy (OR = 1.61, 95% CI = 0.65 to 4.00, *I^2^* = 30%; *P* = .31; n = 467), and targeted therapy (OR = 2.57, 95% CI = 0.93 to 7.09, *I^2^* = 0%; *P* = .07; n = 181). The number of reported patients having undergone hormonal therapy and surgery were not adequate for subgroup analysis. Figure 4 shows the forest plots of mortality outcomes with different types of cancer-directed treatment with COVID-19.

### Cancer Survivors

The definition of cancer survivors was defined variably by different studies. Wang et al. (15) defined cancer survivors as patients treated for cancer within the past 5 years without any active disease. Zhang et al. (16) defined cancer survivors as patients who had not received cancer-directed therapy in the in the past 1 month. Liang et al. (17) defined cancer survivors as patients who had not received cancer-directed therapy recently. Patients receiving active cancer-directed therapy were at higher risk of developing severe disease compared with patients classified as cancer survivors (OR = 2.58, 95% CI = 1.35 to 4.95, *I^2^* = 0%; *P* = .004; n = 366).

### Ongoing Cancer-Directed Therapy Clinical Trials in COVID-19 Patients

Currently, only 12 active ongoing clinical trials are assessing interventions specific to cancer patients. This represents only 0.7% of all the ongoing treatment-based studies (18). The interventions being assessed include the chloroquine analog (GNS561), an anti PD-1 antibody (nivolumab); an anti-interleukin-6 receptor (tocilizumab); an anti-interleukin-8 (BMS-986253), a SUMOylation inhibitor (TAK-981), ivermectin with losartan, lopinavir/ritonavir, rintatolimod and IFN alpha-2b, hydroxychloroquine, TL-895, radiation and azithromycin. The chloroquine analog and azithromycin are being tested as prophylactic agents, whereas the others are being evaluated as therapeutic agents (Supplementary Table 2, available online).

## Discussion 

This systematic review and meta-analysis included 181 323 patients from 26 studies involving 23 736 cancer patients affected by COVID-19. Our meta-analysis shows that cancer patients with COVID-19 have an increased likelihood of death compared with noncancer COVID-19 patients, which was particularly driven by patients in China. Cancer patients were more likely to be intubated than noncancer patients, but ICU admission rates were not statistically significant between the 2 groups. Hematological malignancies were associated with the highest mortality, followed by lung cancer. There was no association between receipt of a particular type of oncologic therapy and mortality.

Some of our findings are rather intuitive; nonetheless, having objective data to confirm our suspicions is helpful in making evidence-based recommendations for deployment of limited resources within health-care environments. For instance, the higher mortality in patients with hematological malignancies could be readily explained by the greater degree of immunosuppression used in the treatment of these patients, even in the absence of bone marrow or stem cell transplantation. The underlying immunosuppressive microenvironment due to dysfunctional immune cell production is a fundamental attribute of the malignancies that drives their pathogenesis (19). Faced with an added insult (e.g., COVID-19), these patients may not have adequate immune reserves to combat the infection. In a flow cytometric analysis of 522 patients from China, T-cell lymphopenia was an important prognostic factor for mortality in COVID-19 patients (20). The OpenSAFELY Collaborative from the United Kingdom published one of the largest epidemiological reports, stating that the hazard ratio (HR) of death in patients with hematological malignancies was 3.52 (95% CI = 2.41 to 5.14) as compared with 1.56 (95% CI = 1.29 to 1.89) for solid tumors (7). 

Our investigation did not show any clear association between treatment modality and mortality, which is reassuring. There have been concerns regarding an association between immune checkpoint inhibitors and the hyperactive phase of COVID-19 infection. In an analysis of 67 consecutive lung cancer patients with COVID-19 treated with PD-1 blockade, no statistically significant association was found between the timing of PD-1 blockade and COVID-19 severity as well as mortality (9). We observed a numerical increase in the odds of death from targeted therapy and chemotherapy, but it was not statistically significant. However, there was a lack of information regarding the particular agent or class; hence, we were not able to perform corresponding subgroup analyses. Of note, no association was found between radiation delivery and COVID-19. Radiation is known to deplete the circulating as well as resident lymphocyte subpopulations, and this lymphopenia is known to last months to years (8). However, these results should also be interpreted with caution in view of the lack of robust numbers in the groups analyzed.

A few caveats about our analysis are noteworthy. The pooled mortality rates in such a meta-analysis may be misleading given that cancer patients are often older and have more comorbidities. Hence, the actual magnitude of mortality in cancer patients with COVID-19 using age-matched cohorts might be lower than reported in these studies. Additionally, mortality differences seemed to be driven by Chinese patients, which could imply unforeseen COVID-19 treatment-related effects or genetic polymorphisms as compared with Western populations. Within Western populations, the findings of our analysis may still help inform how resources are redeployed within oncology units or cancer centers. For instance, the greater mortality among patients with hematological malignancies may argue for marshalling additional resources to these units in a cancer center. It may also support a conscious decision to delay highly immunosuppressive treatments such as bone marrow transplantation, stem cell transplantation, or chimeric antigen receptor T cells (CAR-T cell) therapy. However, modifying standard therapeutic protocols to accommodate predicted disparities in mortality engenders additional risks in terms of disease progression. Clearly, such modifications should be guided by a nuanced risk-benefit analysis based on the best available data.

As is customary with such data, the results should be interpreted cautiously, because the studies were heterogeneous and sample sizes were variable. The majority of studies were single-institutional series with selective reporting of data and high publication bias. The contribution of age, comorbidities, preexisting immunodeficiency, and polypharmacy to mortality outcomes in cancer patients with COVID-19 remains difficult to assess. There is lack of data on individual reasons of mortality. Furthermore, none of the studies reported mortality outcomes in relation to COVID-19–directed therapy. Accordingly, the potential interactions between cancer-directed therapy and COVID-19–directed therapy were not documented in any of the studies (21). Taken together, these attributes make the conclusions arising from our meta-analysis predominately hypothesis generating rather than definitive and/or conclusive. However, at this point in time, these studies remain the core of the limited evidence on this topic to date, and higher quality of studies in the future may allow for corresponding improvements to future meta-analyses.

### Strengths of the Study

The first meta-analysis to assess the outcomes of cancer patients affected by COVID-19 and comparing it with noncancer patients. We also have assessed the potential interactions between different types of cancer-directed therapies and its potential impact on COVID-19 outcomes. The prognosis of different subtypes of cancer as well as geographical differences in the outcomes have also been assessed. We feel this is incredibly important and timely piece of research with the potential to influence policy throughout the pandemic. Furthermore, it provides a foundation upon which further research can be built as more and more data becomes publicly available.

### Implications for Clinical Practice

Cancer patients with COVID-19 have a higher probability of severe disease, increased ventilatory requirements, and mortality compared with the general population. Patients with hematological malignancies and lung cancer are at increased risk of death compared with other subtypes of cancer. There is a need for prospective registration of cancer patients with COVID-19 in registry initiatives like the UK Coronavirus Cancer Monitoring Project and the American Society of Clinical Oncology (ASCO) Survey on COVID-19 in Oncology Registry (ASCO Registry) for better understanding of the impact of cancer-directed therapies as well as COVID-directed therapies on mortality and morbidity outcomes (22).

## Funding

None.

## Notes

**Role of the funder:** Not applicable.

**Disclosures:** There are no conflicts of interest to disclose related to this article

**Author contributions:** Conception and design: BhanuPrasad Venkatesulu, Viveksandeep Thoguluva Chandrasekar, Prashanth Girdhar. Acquisition of data: BhanuPrasad Venkatesulu, Viveksandeep Thoguluva Chandrasekar, Prashanth Girdhar. Analysis and interpretation of data: BhanuPrasad Venkatesulu, Viveksandeep Thoguluva Chandrasekar, Prashanth Girdhar. Drafting the article or revising it critically for important intellectual content: BhanuPrasad Venkatesulu, Viveksandeep Thoguluva Chandrasekar, Prashanth Girdhar, Pragati Advani, Amrish Sharma, Thiraviyam Elumalai, Cheng En Hsieh, Hagar I. Elghazawy, Vivek Verma, Sunil Krishnan. Final approval of the version to be submitted: BhanuPrasad Venkatesulu, Viveksandeep Thoguluva Chandrasekar, Prashanth Girdhar, Pragati Advani, Amrish Sharma, Thiraviyam Elumalai, Cheng En Hsieh, Hagar I. Elghazawy, Vivek Verma, Sunil Krishnan.

**Acknowledgments:** We thank the researchers who posted the studies in the public domain.

## Data Availability

The data used for analysis can be provided upon due request to the corresponding author. 

**Table 1.** Summary of the studies included in the meta-analysis^a^

Study	Study design	Country	Median age, y	No. and/or % of patients with specific Cancer subtypes	Comments
Dai et al. 2020 (23)	Retrospective; multicenter (n = 105)	China	64	Lung = 20.95%; GI = 12.38%; breast = 10.48%; thyroid = 10.48%; hematologic = 8.57%	Death rates (OR= 2.34, 95% CI = 1.15 to 4.77; *P* = .03), ICU admission (OR = 2.84, 95% CI = 1.59 to 5.08; *P* < .01), severe or critical symptom (OR = 2.79, 95% CI = 1.74 to 4.41; *P* < .01).
He et al. 2020 (24)	Retrospective; multicenter (n = 13)	China	35	AML = 4; ALL = 5; plasma cell myeloma = 3; MDS = 1	8 of 13 with hematological cancers and COVID-19 died. Need for mechanical ventilation: 1 of 13
Joharatnam-Hogan et al. 2020 (25)	Retrospective; multicenter (n = 26)	UK	76	Colorectal = 5; prostate cancer = 5; others = 16	3 of 26 of cancer patients received noninvasive ventilatory support, all of whom died; 3 of 6 who died received chemotherapy; and 3 of 6 who died received targeted treatment.
Yu et al. 2020 (26)	Retrospective; single center (n = 12)	China	66	NSCLC = 7; colorectal = 2; pancreatic = 1; breast = 1; urothelial = 1	3 of 12 patients died; 2 of 7 NSCLC died, 1 of 1 pancreatic cancer died, 1 patient on chemo radiotherapy, 1 patient on follow-up after treatment, and 1 patient on best supportive care died.
Liang et al. 2020 (17)	Retrospective, cancer database China (n = 18)	China	63	Lung = 5	Cancer patients had higher risk of severe events compared with patients without cancer (9 [50%] of 18 patients vs 245 [16%] of 1572 patients; *P* = .0008).
Luo et al. 2020 (9)	Retrospective; single center (n = 69)	USA	69	Lung = 69	Mortality rate: 16 of 67 (24%); rate of severe disease a composite of ICU/intubation/transition to DNI status: 24 of 65 (37%); rate of hospitalization: 42 of 67 (63%).
Mehta et al. 2020 (6)	Retrospective; single center (n = 218)	USA	69	GU = 39; breast = 24; colorectal = 13; gynecologic = 8; lung = 5; head and neck = 7; neuro = 7; hematologic = 34	61 of 218 (28%) patients expired. The mortality was 25% of all solid tumor patients and was seen to occur at higher rates in lung cancers (55%) and hematologic malignancies (37%). Active chemotherapy and radiation therapy treatment were not associated with increased case fatality.
Miyashita et al. 2020 (27)	Retrospective; single center (n = 334)	USA	NA	Breast = 57; prostate = 56; lung = 23; urothelial = 18; colon = 16	37 of 334 cancer patients intubated and died; 314 of 5354 noncancer patients intubated, and 518 patients died.
Montopoli et al. 2020 (28)	Retrospective; multicenter (n = 786)	Italy	NA	Prostate = 118; GU = 73; colorectal cancer = 65; hematological = 47	47.0% (n = 2131) of male patients were hospitalized, and 6.9% (n = 312) died, and among patients with cancer, 67.9% (n = 292) were hospitalized, and 17.4% (n = 75) died. 0 of 4 patients infected with COVID-19 on anti-androgen therapy (ADT) died; 18 of 114 (16%) prostate cancer patients not on ADT died.
Martin-Moro et al. 2020 (29)	Retrospective; single center (n = 34)	Spain	72.5	Acute leukemia = 7; MDS = 3; CLL = 6; lymphoma = 6; plasma cell dyscrasia = 7	11 (32%) of 34 died. Acute leukemia: 4 of 7 (57%) patients died; MDS: 3 of 3 patients died; plasma cell dyscrasia: 1 of 7 (14%) patients died; myeloproliferative neoplasm: 3 of 5 (60%) patients died.
Robilotti et al. 2020 (30)	Retrospective; single center (n = 423)	USA	NA	Hematologic = 103 (25%); breast = 86 (20%); colorectal = 37 (9%); lung = 35 (8%); prostate = 26 (6%)	Death: 39 of 423 (9%); mechanical ventilation: 39 (9%). Treatment with immunotherapy also remained an independent predictor of severe respiratory illness. Lung cancer severe illness: 15 of 35 (43%); others severe illness: 39 of 240 (16%). Severe illness in immunotherapy: 12 of 31 (39%) Severe illness in non-immunotherapy: 42 of 244 (17%)
Vuagnat et al. 2020 (31)	Prospective; cohort; single center (n = 59)	France	58	Breast	28 of 59 patients (47%) were hospitalized. No association between prior radiation therapy fields or extent of radiation therapy sequelae and extent of COVID-19 lung lesions.
Wang et al. 2020 (15)	Retrospective; multicenter (n = 283)	China	63	Lung = 51 (18%); breast = 38 (13%); colorectal = 34 (12%)	Overall mortality rate was 18% (50 of 283). The current cancer patients exhibited worse outcomes vs former cancer patients (overall survival, HR = 2.45, 95% CI = 1.10 to 5.44; *P* = .02; mortality rate, 21% vs 9%). Higher mortality rate observed in lymphohematopoietic malignancies (53%, 9 of 17). The highest mortality rate observed in patients receiving recent chemotherapy (33%), followed by surgery (26%), other antitumor treatments (19%), and no antitumor treatment (15%).
Yang et al. 2020 (32)	Retrospective; single center (n = 52)	China	63	Lung = 19.2%; breast = 17.3%; rectal = 15.4%; colon = 9.6%; cervical = 7.7%; thyroid = 5.8%	10 (19.2%) patients received anticancer treatment within 1 month. 11 severe or critical patients died in this study; 2 of 11 received chemotherapy; 1 had undergone surgery.
Zhang et al. 2020 (16)	Retrospective; multicenter (n = 67)	China	66	Lung = 15	23 (34.3%) patients had received anticancer treatment recently; 18 (26.9%) patients died from COVID-19. Lung cancer accounted for the highest proportion of COVID-19–resulted deaths (33.3%, 5 of 15); digestive system cancers (5 of 17, 29.4%) died.
Zhang et al. 2020 (33)	Retrospective; multicenter (n = 28)	China	65	Lung = 7; oesophageal = 4; breast cancer = 3; head and neck = 3	15 (53.6%) patients had severe events, and the mortality rate was 28.6%. If the last antitumor treatment was within 14 days, it statistically significantly increased the risk of developing severe events (HR = 4.079, 95% CI = 1.086 to 15.322; *P* = .037).
Stroppa et al. 2020 (34)	Retrospective; single center (n = 25)	Italy	71	Lung = 8; GI tumor = 6; GU = 6; breast = 2, hematologic = 2; undefined site = 1	12 (48%) patients were treated with anticancer therapy: 8 (66.67%) with chemotherapy and 4 (33.33%) with immunotherapy; 9 of 25 patients died; 2 breast cancer patients, 3 GU patients, 2 hematological malignancy patients, and 2 lung cancer patients died.
Basse et al. 2020 (35)	Retrospective; single center (n = 141)	France	62	Breast = 40%; lung = 13%; hematological = 13%; gynecological = 9%	26 (18%) died from COVID-19. Lung cancers had the worst outcome (n = 6, ie, 23% of all deaths) followed by hematological malignancies (n = 5; 19%), breast (n = 5, 19%), and gynecological cancers (n = 2; 7%).
Russell et al. 2020 (36)	Retrospective; single center (n = 106)	UK	67	Urological/gynecological = 34%; hematological = 18%; breast = 15%	14 cancer patients died of COVID-19 (13%).
Rafi Kabaritti et al. 2020 (37)	Retrospective; single center (n = 107)	USA	70	Breast = 28; prostate = 27; lung = 14	24 patients died; lung cancer diagnosis was also associated with increased risk of death (HR = 2.96, 95% CI = 1.09 to 9.27; *P* = .034). Cox proportional hazards models demonstrated a statistically significant association between mean radiotherapy dose delivered to the lungs and risk of death (HR = 1.12 per Gy, 95% CI = 1.04 to 1.20; *P* = .002).
Deng et al. 2020 (38)	Retrospective; database (n = 107)	China	NA	NA	6 cancer patients died (5.6%); overall mortality rate: 2.3%.
Ma et al. 2020 (39)	Retrospective; single center (n = 37)	China	62	Colorectal = 29.7%; lung = 21.6%; breast = 18.9%	5 (13.5%) cancer patients died.
Cook et al. 2020 (40)	Retrospective (n = 75)	UK	73	Multiple myeloma	41 of 73 patients died.
Kalinsky et al. 2020 (41)	Retrospective (n = 27)	USA	56	Breast cancer	16 (59%) received chemotherapy, 12 (44%) hormone therapy, 6 (22%) HER2-directed therapy, 1 (4%) checkpoint inhibitor, 6 (22%) breast surgery, and 2 (7%) radiation therapy. 1 of 27 patients died.
Gonzalez-Cao et al. 2020 (42)	Retrospective (n = 50)	Spain	69	Malignant melanoma	Mortality rates from COVID-19 according to melanoma treatment type were 16%, 15%, and 36% for patients on immunotherapy and targeted drugs and for those who were not undergoing active cancer treatment, respectively.
Fratino et al. 2020 (43)	Database retrospective multicenter (n = 20 534)	Italy	NA	NA	150 of 20 534 patients died of COVID-19 disease.

a ALL = acute lymphocytic leukemia; AML = acute myeloid leukemia; CI = confidence interval; DNI = do not intubate; GI = gastrointestinal; GU = genitourinary; HR = hazard ratio; NA = not applicable; NSCLC = non–small cell lung cancer; MDS = myelodysplastic syndrome; OR = odds ratio.

## Supplementary Material

pkaa102_Supplementary_Data
