# Supplementary material for: A Systematic Review and Meta-Analysis of Cancer Patients Affected by a Novel Coronavirus
Source: JNCI Cancer Spectr. 2021 Feb 24;5(2):pkaa102. doi: 10.1093/jncics/pkaa102 (PMC7928783; doi:10.1093/jncics/pkaa102)
Supplement: pkaa102_Supplementary_Data [file pkaa102_supplementary_data.pdf]

SUPPLEMENTARY MATERIALS

Supplementary Methods

PRISMA Checklist for Systematic Reviews

Section/topic	#	Checklist item	Reported on page #
**TITLE**			
Title	1	Identify the report as a systematic review, meta-analysis, or both.	1
**ABSTRACT**			
Structured summary	2	Provide a structured summary including, as applicable: background; objectives; data sources; study eligibility criteria, participants, and interventions; study appraisal and synthesis methods; results; limitations; conclusions and implications of key findings; systematic review registration number.	1
**INTRODUCTION**			
Rationale	3	Describe the rationale for the review in the context of what is already known.	
Objectives	4	Provide an explicit statement of questions being addressed with reference to participants, interventions, comparisons, outcomes, and study design (PICOS).	2
**METHODS**			
Protocol and registration	5	Indicate if a review protocol exists, if and where it can be accessed (e.g., Web address), and, if available, provide registration information including registration number.	3
Eligibility criteria	6	Specify study characteristics (e.g., PICOS, length of follow-up) and report characteristics (e.g., years considered, language, publication status) used as criteria for eligibility, giving rationale.	4
Information sources	7	Describe all information sources (e.g., databases with dates of coverage, contact with study authors to identify additional studies) in the search and date last searched.	3
Search	8	Present full electronic search strategy for at least one database, including any limits used, such that it could be repeated.	3
Study selection	9	State the process for selecting studies (i.e., screening, eligibility, included in systematic review, and, if applicable, included in the meta-analysis).	4
Data collection process	10	Describe method of data extraction from reports (e.g., piloted forms, independently, in duplicate) and any processes for obtaining and confirming data from investigators.	4
Data items	11	List and define all variables for which data were sought (e.g., PICOS, funding sources) and any assumptions and simplifications made.	4
Risk of bias in individual studies	12	Describe methods used for assessing risk of bias of individual studies (including specification of whether this was done at the study or outcome level), and how this information is to be used in any data synthesis.	Heterogeneity described
Summary measures	13	State the principal summary measures (e.g., risk ratio, difference in means).	4,5
Synthesis of results	14	Describe the methods of handling data and combining results of studies, if done, including measures of consistency (e.g., I^2) for each meta-analysis.	4,5

Section/topic	#	Checklist item	Reported on page #
Risk of bias across studies	15	Specify any assessment of risk of bias that may affect the cumulative evidence (e.g., publication bias, selective reporting within studies).	Yes
Additional analyses	16	Describe methods of additional analyses (e.g., sensitivity or subgroup analyses, meta-regression), if done, indicating which were pre-specified.	5
**RESULTS**			
Study selection	17	Give numbers of studies screened, assessed for eligibility, and included in the review, with reasons for exclusions at each stage, ideally with a flow diagram.	5
Study characteristics	18	For each study, present characteristics for which data were extracted (e.g., study size, PICOS, follow-up period) and provide the citations.	5
Risk of bias within studies	19	Present data on risk of bias of each study and, if available, any outcome level assessment (see item 12).	no
Results of individual studies	20	For all outcomes considered (benefits or harms), present, for each study: (a) simple summary data for each intervention group (b) effect estimates and confidence intervals, ideally with a forest plot.	Figure 2,3,4
Synthesis of results	21	Present results of each meta-analysis done, including confidence intervals and measures of consistency.	Figure 2,3,4
Risk of bias across studies	22	Present results of any assessment of risk of bias across studies (see Item 15).	Retrospective data
Additional analysis	23	Give results of additional analyses, if done (e.g., sensitivity or subgroup analyses, meta-regression [see Item 16]).	Yes
**DISCUSSION**			
Summary of evidence	24	Summarize the main findings including the strength of evidence for each main outcome; consider their relevance to key groups (e.g., healthcare providers, users, and policy makers).	8,9,10
Limitations	25	Discuss limitations at study and outcome level (e.g., risk of bias), and at review-level (e.g., incomplete retrieval of identified research, reporting bias).	11
Conclusions	26	Provide a general interpretation of the results in the context of other evidence, and implications for future research.	11
**FUNDING**			
Funding	27	Describe sources of funding for the systematic review and other support (e.g., supply of data); role of funders for the systematic review.	None

Search strategy used for the systematic review

Search strategy used for searching PubMed, Embase and Cochrane central

Search strategy for PubMed

1. Coronavirus OR COVID-19 OR SARS-CoV-2 OR novel coronavirus
2. Tumor OR cancer OR malignancy OR neoplasm

Search strings attached:

3 #**1 AND #2**

((((("coronavirus"[MeSH Terms] OR "coronavirus"[All Fields]) OR "coronaviruses"[All Fields]) OR ((((((("covid 19"[All Fields] OR "covid 2019"[All Fields]) OR "severe acute respiratory syndrome coronavirus 2"[Supplementary Concept]) OR "severe acute respiratory syndrome coronavirus 2"[All Fields]) OR "2019 ncov"[All Fields]) OR "sars cov 2"[All Fields]) OR "2019ncov"[All Fields]) OR (("wuhan"[All Fields] AND ("coronavirus"[MeSH Terms] OR "coronavirus"[All Fields])) AND (2019/12/1:2019/12/31[Date - Publication] OR 2020/1/1:2020/12/31[Date - Publication])))) OR (("severe acute respiratory syndrome coronavirus 2"[Supplementary Concept] OR "severe acute respiratory syndrome coronavirus 2"[All Fields]) OR "sars cov 2"[All Fields])) OR ((("novel"[All Fields] OR "novel s"[All Fields]) OR "novels"[All Fields]) AND (("coronavirus"[MeSH Terms] OR "coronavirus"[All Fields]) OR "coronaviruses"[All Fields]))) AND ((((((((((((((((((((("cysts"[MeSH Terms] OR "cysts"[All Fields]) OR "cyst"[All Fields]) OR "neoplasm s"[All Fields]) OR "neoplasms"[MeSH Terms]) OR "neoplasms"[All Fields]) OR "neoplasm"[All Fields]) OR "neurofibroma"[MeSH Terms]) OR "neurofibroma"[All Fields]) OR "neurofibromas"[All Fields]) OR "tumor s"[All Fields]) OR "tumoral"[All Fields]) OR "tumorous"[All Fields]) OR "tumour"[All Fields]) OR "tumor"[All Fields]) OR "tumour s"[All Fields]) OR "tumoural"[All Fields]) OR "tumourous"[All Fields]) OR "tumours"[All Fields]) OR "tumors"[All Fields]) OR ((((((((("cancer s"[All Fields] OR "cancerated"[All Fields]) OR "canceration"[All Fields]) OR "cancerization"[All Fields]) OR "cancerized"[All Fields]) OR "cancerous"[All Fields]) OR "neoplasms"[MeSH Terms]) OR "neoplasms"[All Fields]) OR "cancer"[All Fields]) OR "cancers"[All Fields])) OR ((((((((((((("malign"[All Fields] OR "malignance"[All Fields]) OR "malignances"[All Fields]) OR "malignant"[All Fields]) OR "malignants"[All Fields]) OR "malignities"[All Fields]) OR "malignity"[All Fields]) OR "malignization"[All Fields]) OR "malignized"[All Fields]) OR "maligns"[All Fields]) OR "neoplasms"[MeSH Terms]) OR "neoplasms"[All Fields]) OR "malignancies"[All Fields]) OR "malignancy"[All Fields])) OR ((((((((((((((((((("cysts"[MeSH Terms] OR "cysts"[All Fields]) OR "cyst"[All Fields]) OR "neoplasm s"[All Fields]) OR "neoplasms"[MeSH Terms]) OR "neoplasms"[All Fields]) OR "neoplasm"[All Fields]) OR "neurofibroma"[MeSH Terms]) OR "neurofibroma"[All Fields]) OR "neurofibromas"[All Fields]) OR "tumor s"[All Fields]) OR "tumoral"[All Fields]) OR "tumorous"[All Fields]) OR "tumour"[All Fields]) OR "tumor"[All Fields]) OR "tumour s"[All Fields]) OR "tumoural"[All Fields]) OR "tumourous"[All Fields]) OR "tumours"[All Fields]) OR "tumors"[All Fields])) 2,055 17:25:08

2 **Tumor OR cancer OR malignancy OR neoplasm**

"cysts"[MeSH Terms] OR "cysts"[All Fields] OR "cyst"[All Fields] OR "neoplasm s"[All Fields] OR "neoplasms"[MeSH Terms] OR "neoplasms"[All Fields] OR "neoplasm"[All Fields] OR "neurofibroma"[MeSH Terms] OR "neurofibroma"[All Fields] OR "neurofibromas"[All Fields] OR "tumor s"[All Fields] OR "tumoral"[All Fields] OR "tumorous"[All Fields] OR "tumour"[All Fields] OR "tumor"[All Fields] OR "tumour s"[All Fields] OR "tumoural"[All Fields] OR "tumourous"[All Fields] OR "tumours"[All Fields] OR "tumors"[All Fields] OR "cancer s"[All Fields] OR "cancerated"[All Fields] OR "canceration"[All Fields] OR "cancerization"[All Fields] OR "cancerized"[All Fields] OR "cancerous"[All Fields] OR "neoplasms"[MeSH Terms] OR "neoplasms"[All Fields] OR "cancer"[All Fields] OR "cancers"[All Fields] OR "malign"[All Fields] OR "malignance"[All Fields] OR "malignances"[All Fields] OR "malignant"[All Fields] OR "malignants"[All Fields] OR "malignities"[All Fields] OR "malignity"[All Fields] OR "malignization"[All Fields] OR "malignized"[All Fields] OR "maligns"[All Fields] OR "neoplasms"[MeSH Terms] OR "neoplasms"[All Fields] OR "malignancies"[All Fields] OR "malignancy"[All Fields] OR "cysts"[MeSH Terms] OR "cysts"[All Fields] OR "cyst"[All Fields] OR "neoplasm s"[All Fields] OR "neoplasms"[MeSH Terms] OR "neoplasms"[All Fields] OR "neoplasm"[All Fields] OR "neurofibroma"[MeSH Terms] OR "neurofibroma"[All Fields] OR "neurofibromas"[All Fields] OR "tumor s"[All Fields] OR "tumoral"[All Fields] OR "tumorous"[All Fields] OR "tumour"[All Fields] OR "tumor"[All Fields] OR "tumour s"[All Fields] OR "tumoural"[All Fields] OR "tumourous"[All Fields] OR "tumours"[All Fields] OR "tumors"[All Fields] 4,694,983 17:24:48

1 **Coronavirus OR COVID-19 OR SARS-CoV-2 OR novel coronavirus**

(((("coronavirus"[MeSH Terms] OR "coronavirus"[All Fields]) OR "coronaviruses"[All Fields]) OR ((((((("covid 19"[All Fields] OR "covid 2019"[All Fields]) OR "severe acute respiratory syndrome coronavirus 2"[Supplementary Concept]) OR "severe acute respiratory syndrome coronavirus 2"[All Fields]) OR "2019 ncov"[All Fields]) OR "sars cov 2"[All Fields]) OR "2019ncov"[All Fields]) OR (("wuhan"[All Fields] AND ("coronavirus"[MeSH Terms] OR "coronavirus"[All Fields])) AND (2019/12/1:2019/12/31[Date - Publication] OR 2020/1/1:2020/12/31[Date - Publication])))) OR (("severe acute respiratory syndrome coronavirus 2"[Supplementary Concept] OR "severe acute respiratory syndrome coronavirus 2"[All Fields]) OR "sars cov 2"[All Fields])) OR ((("novel"[All Fields] OR "novel s"[All Fields]) OR "novels"[All Fields]) AND (("coronavirus"[MeSH Terms] OR "coronavirus"[All Fields]) OR "coronaviruses"[All Fields])) 31,824 17:24:11

Search strategy for Embase

No	Query	Results	Date
#3	#1 AND #2	2673	23 May 2020
#2	tumor OR cancer OR malignancy OR neoplasm	5,659,261	23 May 2020
#1	'coronavirus'/exp OR coronavirus OR 'covid 19'/exp OR 'covid 19' OR 'sars cov 2' OR 'novel coronavirus' OR (novel AND ('coronavirus'/exp OR coronavirus))	33,302	23 May 2020

Search of Cochrane central

Date Run: 23/05/2020 07:47:22

Comment:

ID Search Hits

#1 Coronavirus OR COVID-19 OR SARS-CoV-2 OR novel coronavirus 413

#2 Tumor OR cancer OR malignancy OR neoplasm 198188

#3 #1 AND #2 26

Supplementary Table 1: Summary table for characteristics of included studies

First author	Study design	Country	Study description and outcomes
Dai et al [1]	Retrospective; multicenter	China	**Total number of COVID positive cancer patients:** 105(Pan cancer study)<br>**Comparator:** 536 age-matched non-cancer patients<br>**Study enrollment time period:** January 1, 2020, to February 24, 2020, from 14 hospitals in Wuhan, China.<br>**Median age**:64.00<br>**Male/female:**57/48<br>**Comorbidities:** Hypertension 30; Cardiovascular disease 12; Diabetes 7; Cerebrovascular disease 5; Chronic kidney disease 6; Chronic liver disease 7.<br>**Outcomes assessed:**<br>Death rates [OR, 2.34; 95% confidence interval (CI), (1.15–4.77); P = 0.03],<br>ICU admission [OR, 2.84; 95% CI (1.59–5.08); P < 0.01], Severe or critical symptom [OR, 2.79; 95% CI, (1.74–4.41); P < 0.01].<br>**Cancer subtypes:**<br>Lung cancer:22 (20.95%); Gastrointestinal cancer:13 (12.38%); Breast cancer: 11 (10.48%); Thyroid cancer :11 (10.48%); Hematologic cancer:9 (8.57%).<br>**Cancer specific mortality rates:**<br>Hematologic cancer including leukemia, lymphoma, and myeloma highest death rate n=9: Death rate [3 (33.33%)] ; ICU admission rate [4 (44.44%)]; severe/critical symptoms [6 (66.67%)], and Utilization of invasive mechanical ventilation [2 (22.22%)].<br><br>Lung cancer n=22: Death rate [4 (18.18%)], ICU admission rate [6 (27.27%)], risks of severe/critical symptoms [11 (50.00%)] and utilization of invasive mechanical ventilation [4 (18.18%)].<br><br>Metastatic cancer (stage IV) had even higher risks of death [OR, 5.58; 95% CI (1.71–18.23); P = 0.01], ICU admission [OR, 6.59; 95% CI (2.32–18.72);<br>P < 0.01], having severe conditions [OR, 5.97; 95% CI (2.24– 15.91); P < 0.01], and use of invasive mechanical ventilation<br><br>**Cancer Treatments**<br>13 (12.26%) had radiotherapy, 17 (14.15%) received chemotherapy, 8 (7.62%) received surgery, 4 (3.81%) had targeted therapy, and 6 (5.71%) had immunotherapy within 40 days before the onset of COVID-19 symptoms.<br>Targeted drugs were EGFR–tyrosine kinase inhibitors for treatment of lung cancer, and all of the immunotherapy drugs were PD-1 inhibitors for the treatment of lung cancer.<br><br>**Treatment specific death rates**:<br>Immunotherapy: High rates of death [2 (33.33%) of 6 patients] and high chances of developing critical symptoms [4 (66.67%) of 6 patients].<br><br>Surgery: Death [2 (25.00%) of 8 patients], ICU admission [3 (37.50%) of 8 patients], severe or critical symptoms [5 (62.50%) of 8 patients], invasive ventilation [2 (25.00%) of 8 patients].<br><br>Radiotherapy: No significant differences in having any severe events.

He et al [2]	Retrospective; multicenter	China	**Total number of COVID positive cancer patients**: 13(hematological malignancies alone)<br>**Comparator:** 115 hematological COVID negative cancer patients<br>**Study enrollment time period**: January 23, 2020, to February 14, 2020<br>**Median age**:35 years<br>**Male/female**:7/6<br>**Comorbidities:** Cardiovascular disease and Cerebrovascular disease:3<br>**Outcomes assessed:**<br>Death rate: 8 subjects with hematological cancers and COVID19 died.<br>Need for mechanical ventilation:1/13<br>**Cancer subtypes:**<br>Acute myeloid leukemia: 4; Acute lymphoblastic leukemia 5; Plasma cell myeloma 3; Myelodysplastic syndromes 1
Hogan et al[3]	Retrospective; multicenter	UK	**Total number of COVID positive cancer patients**: 26 (Admitted in 4 North London hospitals)<br>**Comparator:** 26 non-cancer COVID positive patients<br>**Study enrolment time period:** March 12 2020 to April 7 2020<br>**Median age**<br>Cancer patients: 76 years (IQR: 72 – 78)<br>Non-cancer patients: 58 years (IQR: 52 – 77)<br>**Male female ratio**<br>Cancer patients: 15/26 (58% males)<br>Non-cancer patients: 16/26 (62% males)<br>**Co-morbidities:** Atleast 1 co-morbidity in all patients<br>**Outcomes assessed:** Median hospital stay; Patients requiring ventilatory support; Mortality rates of cancer and non-cancer patients; Cancer specific mortality rate<br>**Cancer subtypes:** Colorectal cancer: 5 patients; Prostate cancer: 5 patients; Others: 16 patients<br>**Cancer Treatments:** 12 of 26 patients had received chemotherapy; 7 of 26 patients received targeted therapy; 1 patient received radiotherapy; 1 patient had undergone surgery; No patient received immunotherapy; 16 patients (62%) had received anti-cancer treatment within 4 weeks of admission<br>**Outcomes:** The median hospital stay for patients in both cohorts 7 days. No cancer patient admitted to ITU or treated with invasive ventilation. 12% (3/26) of cancer patients received non-invasive ventilatory support, all of whom died. Overall, 6 cancer patients 6 non-cancer patients died<br>**Cancer specific mortality rates:**<br>4 of 5 prostate cancer patients died, 1 pancreatic and 1 extensive small cell lung cancer patient died<br>**Treatment specific death rates:**<br>50% (3/6) patients who died received chemotherapy, and 50% (3/6) patients who died received targeted treatment
Jing et al [4]	Retrospective; single center	China	**Total number of COVID positive cancer patients**: 12<br>**Comparator:** Cancer patients without COVID infection (n = 1512); Infection rate: 0.79% (95% CI: 0.3 – 1.2%)<br>**Study enrolment time period:** December 30 2019 to February 17 2020<br>**Median age of COVID positive patients:** 66 years (Range: 48 – 78 years)<br>**Male female ratio**<br>**Co-morbidities:**<br>**Outcomes assessed:** Severe COVID 19 infection rate, mortality rate; Duration of hospitalization

			**Cancer subtypes:** 7 of 12 patients were non-small cell lung cancer; 2 colo-rectal cancer patients; 1 each pancreatic, breast and urothelial cancer<br>**Cancer Treatments:** 5 patients received chemotherapy; 2 patients received immunotherapy and 3 patients received radiotherapy; 1 patient underwent surgery;<br>**Outcomes:** 3 of 12 patients died<br>**Cancer specific mortality rates:** 2 of 7 NSCLC died; 1 of 1 pancreatic cancer died<br>**Treatment specific death rates:** 1 patient on chemoradiotherapy, 1 patient on follow up after treatment and 1 patient on best supportive care died
Liang et al [5]	Retrospective, cancer database china	China	**Total number of COVID positive cancer patients**: 18<br>**Comparator:** 1572 non-cancer COVID positive patients<br>**Study enrolment time period:** Data cut-off date: January 31 2020<br>**Mean age:**<br>Cancer patients: 63.1 years (SD: 12.1 years)<br>Non-cancer patients: 48.7 years (SD: 16.2 years)<br>**Outcomes assessed:** Risk of severe events (composite endpoint defined as the percentage of patients being admitted to the intensive care unit<br>requiring invasive ventilation, or death), mortality rates; Time to severe event occurrence;<br>**Cancer subtypes:** 5 (28%) patients lung cancer<br>**Cancer Treatments:** 2 patients unknown treatment status; 4 (25%) patients on active cancer treatment with chemotherapy or surgery; 12 (75%) patients were cancer survivors on follow up after resection<br>**Outcomes:**<br>Patients with cancer were observed to have a higher risk of severe events compared with patients without cancer (9 [50%] of 18 patients *vs* 245 [16%] of 1572 patients; Fisher's exact p=0.0008)<br>Patients with cancer deteriorated more rapidly than those without cancer (median time to severe events 13 days [IQR 6–15] *vs* 43 days [20–not reached]; p<0.0001;<br>**Cancer specific outcomes:**<br>Patients with lung cancer did not have a higher probability of severe events compared with patients with other cancer types (1 [20%] of 5 patients with lung cancer *vs* 8 [62%] of 13 patients with other types of cancer; p=0.294)<br>**Treatment specific outcomes:**<br>Patients who underwent chemotherapy or surgery in the past month had a numerically higher risk (3 [75%] of 4 patients) of clinically severe events than did those not receiving chemotherapy or surgery (6 [43%] of 14 patients)
Luo et al [6]	Retrospective; single center	USA	**Total number of COVID positive cancer patients**: 69 (Lung cancer patients alone; 41 patients received immunotherapy (PD1 blockade)); 80% (55/69) had active or metastatic lung cancer<br>**Comparator:** 28 patients who did not receive PD 1 blockade<br>**Study enrolment time period:** March 12 2020 to April 13 2020<br>**Median age:** 69 (Range 31- 91)<br>**Male female ratio**<br>48% (33/69) were males and 52% (36/69) were female<br>**Co-morbidities:** COPD 12/69 (17%); Non COPD lung disease 14/69 (20%); Obesity 23/69 (33%); Hypertension 38/69 (55%); Diabetes mellitus 21/69 (30%); Congestive heart failure 5/69 (7%)<br>**Outcomes assessed:** Mortality rate; rate of hospitalization; rate of severe disease a composite of ICU/intubation/transition to DNI-status;<br>**Cancer subtypes:** All lung cancer; 64 NSCLC; 5 SCLC

			**Cancer Treatments:** 41 patients received PD1 blockade<br>**Outcomes:** Mortality rate: 16/67 (24%); rate of severe disease a composite of ICU/intubation/transition to DNI-status: 24/65 (37%); Rate of hospitalization: 42/67 (63%)<br>**Treatment specific outcomes:**<br>The odds ratios for the impact of PD-1 blockade on hospitalization, ICU/intubation/DNI, and death diminished to 1.20 (95% CI, 0.33 to 4.23), 0.83 (95% CI, 0.24-2.82), and 1.13 (95% CI, 0.25-5.03)
Mehta et al [7]	Retrospective; single center	USA	**Total number of COVID positive cancer patients**: 218<br>**Comparator:**<br>Age and sex-matched cohort of 1090 patients at a 5:1 ratio of non-cancer to cancer COVID-19 patients from the same time period and from the same hospital system<br>**Study enrolment time period:** March 18 2020 to April 8 2020<br>**Median age:** 69 years (Range 10 – 92 years)<br>**Male female ratio:** 127 (58%) males and 91 (42%) females<br>**Co-morbidities:** Diabetes mellitus: 80 patients; Hypertension: 147; Chronic lung disease: 62 patients; Chronic kidney disease: 54 patients<br>**Outcomes assessed:** Mortality rate; Cancer specific mortality rates; Treatment specific mortality rates<br>**Cancer subtypes:** Genito-urinary: 39 patients; Breast: 24 patients; Colorectal: 13 patients; Gynecologic: 8 patients; Lung: 5 patients; Head and neck: 7 patients; Neuro: 7 patients; Hematologic: 34 patients<br>**Cancer Treatments:** Radiation: 49 patients; Immunotherapy: 5 patients; Chemotherapy: 42 patients;<br>**Outcomes:**<br>61 of 218 cancer (28%) patients expired.<br>**Cancer specific mortality rates:**<br>The mortality was 25% of all solid tumor patients and was seen to occur at higher rates in lung cancers (55%), gastrointestinal cancers (colorectal (38%), pancreas (67%), upper GI (38%)) and gynecologic malignancies (38%). Hematologic malignancies were associated with higher rate of mortality with COVID-19 (37%). Active disease (< 1 year) and advanced metastatic disease showed a trend for increased mortality, but the association did not achieve statistical significance (p = 0.09 and 0.06, respectively).<br>**Treatment specific death rates:**<br>Active chemotherapy and radiation therapy treatment were not associated with increased case fatality. Very few patients in this cohort were on immunotherapy, and did not show any associations with mortality.
Miyashita et al[8]	Retrospective; single center	USA	**Total number of COVID positive cancer patients**: 334<br>**Comparator:** 5354 non-cancer patients<br>**Study enrolment time period:** March 1 2020 to April 6 2020<br>**Outcomes assessed:** Mortality rate; Intubation rate<br>**Cancer subtypes:** Breast: 57 patients; Prostate: 56 patients; Lung: 23 patients; urothelial: 18 patients; Colon cancer: 16 patients;<br>**Outcomes:** 37 of 334 cancer patients intubated and died; 314 of 5354 non-cancer patients intubated and 518 patients died
Montopoli et al [9]	Retrospective; multicenter	Italy	**Total number of COVID positive cancer patients**: 786<br>**Comparator:** 9280 non-cancer patients<br>**Study enrolment time period:** Data cut-off date: April 1 2020<br>**Male female ratio:**<br>For overall population, 4532 males and 4748 females<br>Cancer patients: 430 males and 356 females

			**Outcomes assessed:** Mortality rate in male patients; Severe disease rates in male patients; Comparison of infection, mortality and severe disease rates between patients on ADT versus patients not on ADT<br>**Cancer subtypes:** Distribution described for males only; Prostate: 118; Genito-urinary: 73; Colorectal cancer: 65; Hematological: 47<br>**Outcomes:**<br>47.0% (n=2131) of male patients were hospitalized and 6.9% (n=312) died, while among patients with cancer 67.9% (n=292) were hospitalized and 17.4% (N=75) died. No significant differences were observed between prostate cancer patients and those affected by other types of cancers<br>**Treatment specific death rates:**<br>0 of 4 patients infected with COVID on ADT died; 18 of 114 (16%) prostate cancer patients not on ADT died
Moro et al [10]	Retrospective; single center	Spain	**Total number of COVID positive cancer patients**: 34 (Only Hematological malignancies)<br>**Comparator:** Single arm<br>**Study enrolment time period:** March 9 2020 to April 17 2020<br>**Median age:** 72.5 years (35- 94)<br>**Male female ratio:** 19 males; 15 females<br>**Co-morbidities:**<br>**Outcomes assessed:** Mortality rate<br>**Cancer subtypes:** Acute leukemia: 7; MDS: 3; CLL: 6; Lymphoma: 6; Plasma cell dyscrasia: 7<br>**Cancer Treatments:** Stem cell transplant: 3 patients<br>**Outcomes:** 11 (32%) of 34 died<br>**Cancer specific mortality rates:**<br>Acute leukemia: 4 of 7 (57%) patients died; MDS: 3 of 3 patients died; Plasma cell dyscrasia: 1 of 7 (14%) patients died; Myeloproliferative neoplasm: 3 of 5 (60%) patients died<br>**Treatment specific death rates:**<br>0 patients of stem cell transplant died
Robilotti et al [11]	Retrospective; single center	USA	**Total number of COVID positive cancer patients**: 423<br>**Comparator:** Single arm<br>**Study enrolment time period:** March 10 2020 to April 7 2020<br>**Male female ratio:** 212 males and 211 females<br>**Co-morbidities:** Hypertension: 214 (50%); Diabetes mellitus: 84 (20%); Asthma: 43 (10%); COPD: 29 (7%)<br>**Outcomes assessed:** Mortality rate; Assess risk factors for hospitalization and severe respiratory illness, defined as the requirement for high-flow oxygen supplementation or mechanical ventilation.<br>**Cancer subtypes:** Hematologic malignancies: 103 (25%); Breast: 86 (20%); Colorectal: 37 (9%); Lung: 35 (8%); Prostate: 26 (6%)<br>**Cancer Treatments:** Chemotherapy: 191 (45%); Immunotherapy: 31 (7%)<br>**Outcomes:**<br>Death: 39 of 423 (9%); Mechanical ventilation: 39 (9%). Severe illness was significantly more common with age > 65 years. Treatment with immunotherapy also remained an independent predictor of severe respiratory illness. Metastatic disease, recent receipt of chemotherapy, or major surgery within the previous 30 days did not show a significant association with either hospitalization or severe respiratory illness<br>**Cancer specific outcomes:**<br>Lung cancer severe illness: 15/ 35 (43%)<br>Others severe illness: 39/ 240 (16%)<br>**Treatment specific outcomes:**<br>Severe illness in immunotherapy: 12/ 31 (39%)

			Severe illness in non-immunotherapy: 42/ 244 (17%)
Vuagnat et al [12]	Prospective; Cohort; Single center	France	**Total number of COVID positive cancer patients**: 59 (Only breast cancer)<br>**Comparator:** Single arm<br>**Study enrolment time period:** March 13 2020 to April 25 2020<br>**Median age:** 58 years (Range: 48 – 68)<br>**Co-morbidities:** Hypertension: 21 (36%); Diabetes mellitus: 10 (17%); Chronic lung disease: 2 (3%)<br>**Outcomes assessed:** Hospitalization rate; Mortality rate; Risk factors for death<br>**Cancer subtypes:** Only Breast cancer<br>**Cancer Treatments:** Surgery: 3 (5%); Chemotherapy: 29 (50%); Radiotherapy: 4 (7%); Endocrine therapy: 19 (32%); Targeted therapy: 13 (22%)<br>**Outcomes:**<br>Twenty-eight of these 59 patients (47%) were hospitalized and 6 (10%) were transferred to an intensive care unit. At the time of analysis, 45/59 (76%) patients were recovering or had been cured, 10/59 (17%) were still followed and 4/59 (7%) had died from COVID-19. All 4 patients who died had significant non-cancer comorbidities. In univariate analysis, hypertension and age (>70) were the two factors associated with a higher risk of intensive care unit admission and/or death<br>**Treatment specific death rates:**<br>No association between prior radiation therapy fields or extent of radiation therapy sequelae and extent of COVID-19 lung lesions.
Wang et al [13]	Retrospective; multicenter	China	**Total number of COVID positive cancer patients**: 283<br>**Comparator:** Single arm<br>**Study enrolment time period:** December 17, 2019 to 86 March 18, 2020<br>**Median age:** 63.0 years [IQR, 55.0 to 70.0]<br>**Male female ratio:** 1:1<br>**Co-morbidities:** The most common comorbidities were hypertension (33%), diabetes (14%), and cardiovascular disease (11%).<br>**Outcomes assessed:** Mortality rate; Assess risk factors for mortality<br>**Cancer subtypes:** 51 (18%) lung cancer, 38 (13%) breast cancer, and 34 (12%) colorectal cancer<br>**Cancer Treatments:** Of the 207 current cancer patients, 95 (46%) have received recent anti-tumor treatments, including 46 (22%) with chemotherapy, 23 (11%) with surgical resection, and 26 (13%) with other anti-tumor treatments.<br>**Outcomes:**<br>The overall mortality rate was 18% (50/283), and the median hospitalization stay for the survivors was 26 days. Amongst all, 76 (27%) were former cancer patients with curative resections for over five years without recurrence. The current cancer patients exhibited worse outcomes versus former cancer patients (overall survival, HR=2.45, 95%CI 1.10 to 5.44, log-rank 96 *p*=0.02; mortality rate, 21% vs 9%)<br>**Cancer specific mortality rates:**<br>Higher mortality rate observed in patients with lymphohematopoietic malignancies (LHM) (53%, 9/17). Multivariable analysis indicated that LHM (*p*=0.001) was one of the independent factors associating with critical 103 illness or death.<br>**Treatment specific death rates:**<br>The highest mortality rate observed in patients receiving recent chemotherapy (33%), followed by surgery (26%), other anti-tumor treatments (19%), and no anti-tumor treatment (15%).
Yang et al [14]	Retrospective; single center	China	**Total number of COVID positive cancer patients**: 52<br>**Comparator:** 1523 non cancer COVID patients<br>**Study enrolment time period:** January 1th to April 15th, 2020

			**Median age**<br>63 years (range from 34 to 98 years)<br>**Male female ratio:** 28 males and 24 females<br>**Co-morbidities:** 33(63.5%) patients had comorbidities, the most common comorbidity was hypertension (17, 51.5%), followed by diabetes (7, 21.2%), coronary heart disease (5, 15.2%), cerebrovascular disease (4, 12.1%), chronic obstructive pulmonary disease (4, 12.1%), chronic hepatitis B (2, 6.1%), cirrhosis (1, 3.0%), chronic renal insufficiency (1, 3.0%) and hypothyroidism (1, 3.0%).<br>**Outcomes assessed:** Mortality rates;<br>**Cancer subtypes: L**ung cancer (19.2%), breast cancer (17.3%), rectal cancer (15.4%), colon cancer (9.6%), cervical cancer (7.7%) and thyroid carcinoma (5.8%**)**<br>**Cancer Treatments:** 10(19.2%) patients received anti-cancer treatment within one month. 6(11.5%) of them received chemotherapy, the remaining 4 patients received one therapy of catheter ablation, resection, pancreaticoduodenectomy, and immunotherapy respectively<br>**Outcomes:**<br>11 severe/critical patients died in this study,<br>**Cancer specific mortality rates:** 2 lung cancer, 2 breast cancer, 2 prostate cancer, 1 gastric cancer, I duodenal cancer, 1 endometrial cancer, 1 ovarian cancer, 1 lymphoma patients died<br>**Treatment specific death rates:**<br>2 of 11 received chemotherapy; 1 had undergone surgery
Zhang H et al [15]	Retrospective; multicenter	China	**Total number of COVID positive cancer patients**: 67<br>**Comparator:** Single arm<br>**Study enrolment time period:** Jan 5, 2020 to Feb 18, 2020<br>**Median age:** 66 years (range: 37-90)<br>**Male female ratio:** 41 males; 26 females<br>**Co-morbidities:** Forty-three (64.2%) patients had other concurrent<br>chronic diseases, of which hypertension (53.7%, n=36), diabetes (19.4%, n=13), and coronary heart disease (11.9%, n=8) were the most common<br>**Outcomes assessed:** Case fatality rate;<br>**Cancer subtypes:** Lung cancer (n=15, 22.4%) was the most common cancer type<br>**Cancer Treatments:** Twenty-three (34.3%) patient had received anticancer treatment recently; Among those 23 patients, 9 cases were receiving chemotherapy-based comprehensive therapies, 7 cases were during perioperative period, 3 cases were receiving supportive treatment, 2 cases were receiving radiotherapy combined with targeted therapy or with endocrine therapy, and the rest 2 patients were receiving endocrinotherapy or immunotherapy.<br>**Outcomes:**<br>18 (26.9%) patients died from COVID-19.<br>**Cancer specific mortality rates:**<br>Lung cancer accounted for the highest proportion COVID-19 resulted deaths (33.3%, 5/15). digestive system cancers (5/17, 29.4%) died;
Zhang L et al [16]	Retrospective; multicenter	China	**Total number of COVID positive cancer patients**: 28<br>**Comparator:** Single arm<br>**Study enrolment time period:** 13 January 2020 to 26 February 2020<br>**Median age:** 65 years (IQR: 56 – 70 years)<br>**Male female ratio:** 17 male; 11 female<br>**Co-morbidities:** Diabetes Mellitus: 4; Cardiovascular disease: 4

			**Outcomes assessed:** Mortality rate; Severe event rate<br>**Cancer subtypes:** Lung cancer was the most frequent cancer type (n: 7; 25.0%); Esophageal cancer: 4; Breast cancer: 3; Head and neck: 3<br>**Cancer Treatments:** Surgery: 21 patients; Chemotherapy: 3; Radiotherapy: 1; immunotherapy: 1<br>**Outcomes:**<br>15 (53.6%) patients had severe events and the mortality rate was 28.6%. If the last anti-tumour treatment was within 14 days, it significantly increased the risk of developing severe events [hazard ratio (HR) 4.079, 95% confidence interval (CI)1.086-15.322, P: 0.037]
Stroppa et al [17]	Retrospective; single center	Italy	**Total number of COVID positive cancer patients**: 25<br>**Comparator:** 31 non cancer COVID patients<br>**Study enrolment time period:** 21 February 2020 and 18 March 2020<br>**Mean age:** Mean age of these patients was 71.64 years (range 50–84 years).<br>**Male female ratio:** 20 males; 5 females<br>**Co-morbidities:**<br>19 (76%), had several comorbidities (in particular diabetes mellitus [32%], hypertension [64%] and chronic obstructive pulmonary disease [28%]).<br>**Outcomes assessed:** Mortality rate<br>**Cancer subtypes:**<br>The most common tumor site was lung (eight patients, 32%) followed by gastroenteric tumor (six patients, 24%), genitourinary tumor (six patients, 24%), breast cancer (two patients, 8%), hematologic tumor (two patients, 8%) and undefined tumor site (one patient, 4%).<br>**Cancer Treatments:**<br>12 (48%) patients were treated with anticancer therapy: eight (66.67%) with chemotherapy and four (33.33%) with immunotherapy<br>**Outcomes:**<br>9 of 25 patients died<br>**Cancer specific mortality rates:** 2 breast cancer patients, 3 genito-urinary patients, 2 hematological malignancy patients, 2 lung cancer patients died
Basse et al[18]	Retrospective; single center	France	**Total number of COVID positive cancer patients**: 141<br>**Comparator:** 9701 non- COVID cancer patients<br>**Study enrolment time period:** March 13 2020 to April 25 2020<br>**Median age:** 62 years<br>**Male female ratio:** Females: 102; Males: 39<br>**Co-morbidities:** Hypertension (34%) and diabetes (17%).<br>**Outcomes assessed:** Mortality rates<br>**Cancer subtypes:** Breast cancer was the most common cancer type (40%), followed by lung cancer (13%), haematological malignancies (13%), gynaecological cancers (9%)<br>**Outcomes:**<br>26 patients (18%) had died from COVID-19<br>**Cancer specific mortality rates:**<br>Lung cancers had the worst outcome (n=6 i.e. 23% of all deaths) followed by haematological malignancies (n=5, 19%), breast (n=5, 19%) and gynaecological cancers (n=2, 7%).
Russels et al [19]	Retrospective; single center	UK	**Total number of COVID positive cancer patients**: 106<br>**Comparator:** Single arm<br>**Study enrolment time period:** 29 February 2020-15 April 2020.

			**Median age:** 67 years<br>**Male female ratio:** 58 males; 48 females<br>**Co-morbidities:** Hypertension was the most commonly reported comorbidity (51%), followed by diabetes mellitus (22%), renal impairment (22%) and cardiovascular disease (20%)<br>**Outcomes assessed:** Mortality rate; Factors associated with mortality<br>**Cancer subtypes:** The most frequently reported tumour types were urological/gynaecological (34%), followed by haematological (18%) and breast (15%)<br>**Outcomes:**<br>14 cancer patients died of COVID-19 (13%)
Kabaritti et al[20]	Retrospective; single center	USA	**Total number of COVID positive cancer patients**: 107<br>**Comparator:** Single arm<br>**Study enrolment time period:** March 14 2020 and April 15 2020<br>**Median age**<br>**Male female ratio:** 53 males; 54 females<br>**Outcomes assessed:** Mortality rate; Risk factors for mortality<br>**Cancer subtypes:** Breast cancer: 28; Prostate cancer: 27; Lung cancer: 14<br>**Outcomes:**<br>24 patients died<br>**Cancer specific mortality rates:**<br>Lung cancer diagnosis was also associated with increased risk of death (HR=2.96, 95% CI: 1.09 to 9.27, p=0.034).<br>**Treatment specific death rates:**<br>Cox proportional hazards models demonstrated a significant association between mean radiotherapy dose delivered to the lungs and risk of death (HR=1.12 per Gy, 95% CI: 1.04 to 1.20, p=0.002).
Deng et al[21]	Retrospective; Database	China	**Total number of COVID positive cancer patients**: 107<br>**Comparator:** 44565 non-cancer COVID patients<br>**Study enrolment time period:** Data cut-off February 11 2020<br>**Outcomes:**<br>6 cancer patients died (5.6%)<br>Overall mortality rate: 2.3%
Ma et al [22]	Retrospective; single center	China	**Total number of COVID positive cancer patients**: 37<br>**Comparator:** Single arm<br>**Study enrolment time period:** January 1, 2020 to March 30, 2020<br>**Median age:** 62 years<br>**Male female ratio:** 20 males; 17 females<br>**Outcomes assessed:** Mortality rates; Severe infection rate<br>**Cancer subtypes:** most common cancer was colorectal<br>cancer (29.7%), followed by lung cancer (21.6%) and breast (18.9%).<br>**Outcomes:**<br>5 (13.5%) cancer patients died
Cook et al [23]	Retrospective	UK	**Total number of COVID positive cancer patients**: 75 (Only multiple myeloma)<br>**Comparator:** Single arm<br>**Study enrolment time period:** Data cut off: 18 May 2020<br>**Median age:** 73 years (range 47-88)<br>**Co-morbidities:** Hypertension was the commonest comorbidity (41.3% of patients)

			**Outcomes assessed:** Mortality rate; Hospital stay; Time to discharge<br>**Outcomes:**<br>41 patients died
Kalinsky et al [24]	Retrospective	USA	**Total number of COVID positive cancer patients**: 27 (Only Breast cancer)<br>**Comparator:** Single arm<br>**Study enrolment time period:** 10 March 2020 to 29 April 2020<br>**Median age:** 56 years (range: 32–87)<br>**Male female ratio:** 26 females; 1 male<br>**Co-morbidities:** Co-morbidities included 15 (56%) with hypertension, 6 (22%) diabetes, and 6 (22%) pulmonary disease.<br>**Outcomes assessed:** Mortality rate; Treatment disruption rate<br>**Cancer Treatments:**<br>16 (59%) received chemotherapy, 12 (44%) hormone therapy, 6 (22%) HER2-directed therapy, 1 (4%) checkpoint inhibitor, 6 (22%) breast surgery, and 2 (7%) radiation therapy<br>**Outcomes:**<br>1 of 27 patients died
Cao et al [25]	Retrospective	Spain	**Total number of COVID positive cancer patients**: 50 (Only melanoma)<br>**Comparator:** Single arm<br>**Study enrolment time period:** April 1 2020 to May 17 2020<br>**Median age:** 69 years (range 6 to 94 years),<br>**Male female ratio:** 27 (54%) patients are males; 23 (46%) are females<br>**Outcomes assessed:** Mortality rates<br>**Cancer Treatments:** Twenty-two (44%) patients were on active anticancer treatment with anti PD-1 antibodies, 16 (32%) patients were on treatment with BRAF plus MEK inhibitors and 12 (24%) patients were not on active cancer treatment<br>**Outcomes:**<br>9 patients died from COVID<br>**Treatment specific death rates:**<br>Mortality rates from COVID-19 according to melanoma treatment type were 16%, 15% and 36% for patients on immunotherapy, targeted drugs, and for those that were not undergoing active cancer treatment, respectively
Fratino et al [26]	Database retrospective multicenter	Italy	**Total number of COVID positive cancer patients**: 20534<br>**Comparator:** Two arms<br>**Study enrolment time period:** Till March 30 2020<br>**Median age:** Median 80.0, Inter Quartile Range—IQR 75.0– 85.8)<br>**Outcomes assessed:** Mortality rates<br>150/20534 patients died of COVID disease

Supplementary Table 2 shows the summary of ongoing clinical trials that are specific to cancer patients with COVID-19 disease

**Study ID**	**Country**	**Study title**	**Intervention assessed**	**Primary outcome**
NCT04333914	France	Prospective Study in Patients with Advanced or Metastatic Cancer and SARS-CoV-2 (COVID-19) Infection	Chloroquine analog (GNS561); anti PD-1 (nivolumab); anti-interleukin-6 receptor (tocilizumab)	28-day survival rate
NCT04347226	USA	Anti-Interleukin-8 (Anti-IL-8) for Cancer Patients With COVID-19	Anti-Interleukin-8BMS-986253	Time to Improvement in the 7-point ordinal scale
NCT03648372	USA	A Study to Evaluate the Safety, Tolerability and Pharmacokinetics (PK) of TAK-981 in Adult Participants with Advanced or Metastatic Solid Tumors or Relapsed/Refractory Hematologic Malignancies and in a Subset with Coronavirus Disease 2019 (COVID-19)	TAK-981	Adverse effects and viral clearance
NCT04370834	USA	Tocilizumab for Patients with Cancer and COVID-19 Disease	Tocilizumab	Frequency of response Length of time from level of care to step down level of care Survival
NCT04369365	Austria	A Single-blinded, Randomized, Placebo Controlled Phase II Trial of Prophylactic Treatment with Oral Azithromycin Versus Placebo in Cancer Patients Undergoing Antineoplastic Treatment During the Corona Virus Disease 19 (COVID-19) Pandemic	Azithromycin	Cumulative number of severe acute respiratory syndrome corona virus 2 (SARS-COV-2) infections
NCT04447235	Brazil	Early Treatment With Ivermectin and LosarTAN for Cancer Patients With COVID-19 Infection (TITAN)	Ivermectin and Losartan	Incidence of severe complications due COVID-19 infection defined as need for ICU admission, need for mechanical ventilation, or death
NCT04455958	USA	Lopinavir/Ritonavir for the Treatment of COVID-19 Positive Patients With Cancer and Immune Suppression in the Last Year	Lopinavir/Ritonavir	Severity of symptoms
NCT04392128	Europe	Study Evaluating the Efficacy of Hydroxychloroquine and Azithromycin in Patients With COVID-19 and Hematological Malignancies (HYACINTHE) (HYACINTHE)	Hydroxychloroquine and Azithromycin	Evaluation of the efficacy of hydroxychloroquine and azithromyncin on the viral load drop at day 5
NCT04379518	USA	Rintatolimod and IFN Alpha-2b for the Treatment of Mild or Moderate COVID-19 Infection in Cancer Patients	Rintatolimod and IFN Alpha-2b	30-day mortality; reduction in hospital stay and ARDS

NCT04381988	USA	A Study of Hydroxychloroquine vs Placebo to Prevent COVID-19 Infection in Patients Receiving Radiotherapy	Hydroxychloroquine	cumulative incidence of SARS-CoV-2 infection
NCT04404361	USA	PRE-VENT Study in Hospitalized Patients With Severe COVID-19 With or Without Cancer	Radiation	Proportion of patients who progress to invasive mechanical ventilation and/or ECMO or death during the 28 days following randomization
NCT04419623	USA	A Study of TL-895 With Standard Available Treatment Versus Standard Available Treatment for the Treatment of COVID-19 in Patients With Cancer	TL-895	To determine the recommended dose of TL-895 to be used in Part 2 based on the observed dose limiting toxicity per dose level; Change in the need for artificial ventilation or death

Supplementary Table 3 shows the demographics of the included populations in the systematic review and meta-analysis

	**Cancer patients with COVID disease**	**Non-Cancer patients with COVID disease**
Total number of patients	23,136 (26 studies)	175,587 (14 studies)
Median age of patients	66 (IQR: 63-69.5) years	53(IQR-36-62) years
Mean age of the patients	65.1± 8.02years	50.3± 11.9 years
Number of Males	1413/2683 – 52.6%	4795/9067 – 52.9%
**Comorbidities**		
Smoker	403/1296 - 31.1%	153/2108 - 7.3%
Hypertension	753/1616 - 46.6%	130/536 - 24.2%
Diabetes mellitus	317/1549 - 20.4%	29/536 - 5.4%
Cardiac disease	510/1458- 34.9%	39/536 - 7.3%
Cerebrovascular disease	53/587 – 9.1%	21/536 – 3.9%
Chronic liver disease	54/587 – 9.2 %	35/536 – 6.5%
Chronic kidney disease	130/1200 – 10.8%	22/536 – 4.1%
Chronic lung disease	210/ 1431 – 14.7%	NA
**Patients symptoms:**		
Fever	1098 / 1573 – 69.8%	421/ 573 – 73.5%
Cough	1033/ 1571 – 65.8%	338/537 – 58.9
Myalgia	18/ 265- 6.8%	38/547 – 6.9 %
Diarrhea	307/ 1367 – 22.5%	2/11- 18%
Headache	19/250 – 7.6%	28/116 – 24.1%
Dyspnea	548/ 250 – 39.8%	55/573 – 9.6%
Fatigue	105/305 – 34.4%	179/ 536 – 33.4%
Treatment given		
Antivirals	512/762 – 67.2%	434/599 – 72.5 %
Antibiotics	474/626 – 75.7%	410/ 599 – 68.4%
Steroids	137/ 396 – 34.6%	96/599 – 16%
Oxygen	132/ 210 – 62.9%	224/ 599 – 37.6%
Dialysis	20/ 233 – 8.6%	3/536 – 5.6%
Extra-corporeal membrane oxygenation	3/ 105 – 2.9%	2/ 536 – 3.7 %

Supplementary Figure 1. Forest plot of outcomes based on Geographical distribution. A) Forest plot of pooled in-hospital all-cause mortality rates between cancer patients and non-cancer patients in studies from Asia. B) Forest plot of pooled in-hospital all-cause mortality rates between cancer patients and non-cancer patients in studies from Europe. C) Forest plot of pooled in-hospital all-cause mortality rates between cancer patients and non-cancer patients in studies from USA. Odds ratio calculated using the Mantel-Haenszel random-effects model.

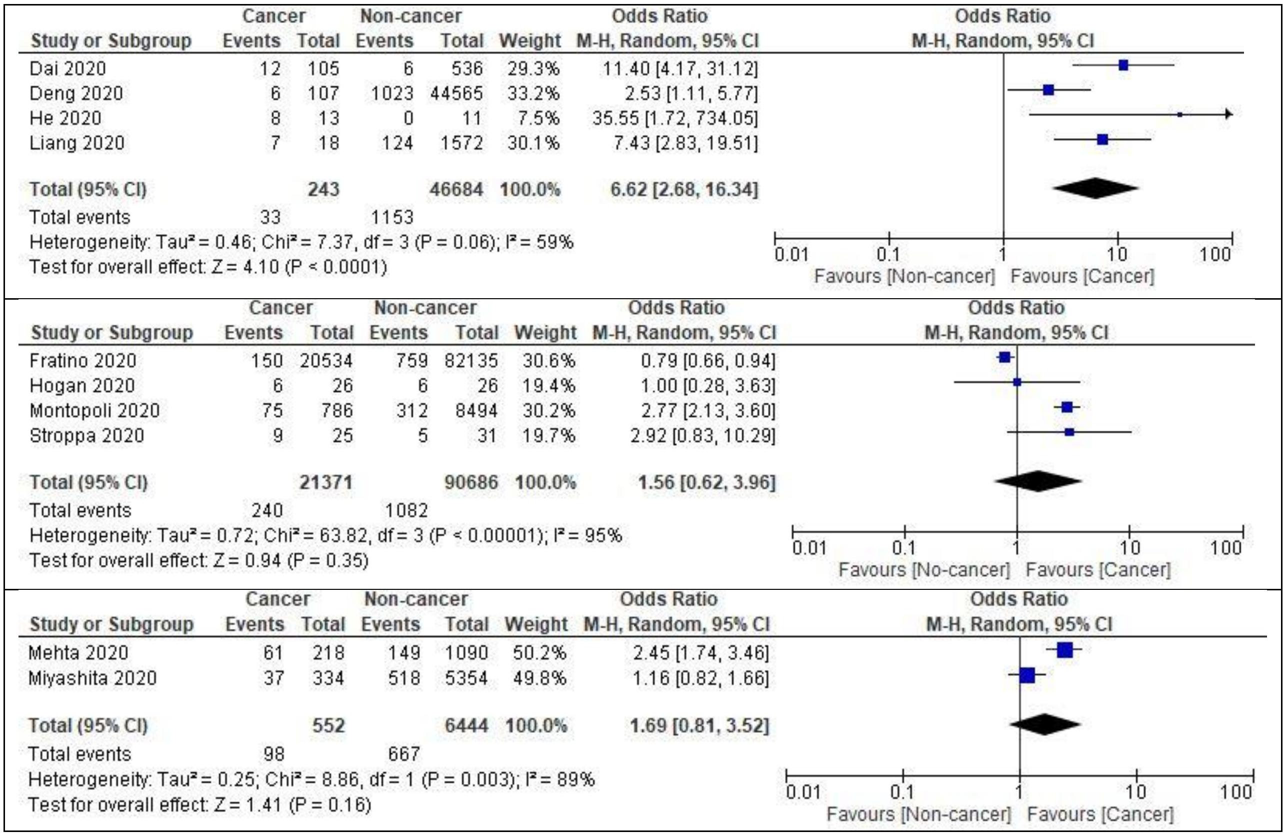

References

[1] Dai M, Liu D, Liu M, Zhou F, Li G, Chen Z, et al. Patients with Cancer Appear More Vulnerable to SARS-COV-2: A Multicenter Study during the COVID-19 Outbreak. Cancer discovery. 2020.

[2] He W, Chen L, Chen L, Yuan G, Fang Y, Chen W, et al. COVID-19 in persons with haematological cancers. Leukemia. 2020:1-9.

[3] Joharatnam-Hogan N, Hochhauser D, Shiu K-K, Rush H, Crolley V, Butcher E, et al. Outcomes of the 2019 Novel Coronavirus in patients with or without a history of cancer - a multi-centre North London experience. 2020:2020.04.16.20061127.

[4] Yu J, Ouyang W, Chua MLK, Xie C. SARS-CoV-2 Transmission in Patients With Cancer at a Tertiary Care Hospital in Wuhan, China. JAMA oncology. 2020.

[5] Liang W, Guan W, Chen R, Wang W, Li J, Xu K, et al. Cancer patients in SARS-CoV-2 infection: a nationwide analysis in China. The Lancet Oncology. 2020;21:335-7.

[6] Luo J, Rizvi H, Egger JV, Preeshagul IR, Wolchok JD, Hellmann MD. Impact of PD-1 blockade on severity of COVID-19 in patients with lung cancers. Cancer discovery. 2020.

[7] Mehta V, Goel S, Kabarriti R, Cole D, Goldfinger M, Acuna-Villaorduna A, et al. Case Fatality Rate of Cancer Patients with COVID-19 in a New York Hospital System. 2020:CD-20-0516.

[8] Miyashita H, Mikami T, Chopra N, Yamada T, Chernyavsky S, Rizk D, et al. Do patients with cancer have a poorer prognosis of COVID-19? An experience in New York City. Annals of oncology : official journal of the European Society for Medical Oncology. 2020.

[9] Montopoli M, Zumerle S, Vettor R, Rugge M, Zorzi M, Catapano CV, et al. Androgen-deprivation therapies for prostate cancer and risk of infection by SARS-CoV-2: a population-based study (n=4532). Annals of oncology : official journal of the European Society for Medical Oncology. 2020.

[10] Martín-Moro F, Marquet J, Piris M, Michael BM, Sáez AJ, Corona M, et al. Survival study of hospitalized patients with concurrent Covid-19 and haematological malignancies. British journal of haematology. 2020.

[11] Robilotti EV, Babady NE, Mead PA, Rolling T, Perez-Johnston R, Bernardes M, et al. Determinants of Severity in Cancer Patients with COVID-19 Illness. 2020:2020.05.04.20086322.

[12] Vuagnat P, Frelaut M, Ramtohul T, Basse C, Diakite S, Noret A, et al. COVID-19 in breast cancer patients: a cohort at the Institut Curie hospitals in the Paris area. 2020:2020.04.30.20085928.

[13] Wang J, Song Q, Chen Y, Wang Z, Chu Q, Gong H, et al. Systematic investigations of COVID-19 in 283 cancer patients. 2020:2020.04.28.20083246.

[14] Yang F, Shi S, Zhu J, Shi J, Dai K, Chen X. Clinical characteristics and outcomes of cancer patients with COVID-19. Journal of medical virology. 2020.

[15] Zhang H-Y, Wang L-W, Chen Y-Y, Shen X-K, Wang Q, Yan Y-Q, et al. A Multicentre Study of 2019 Novel Coronavirus Disease Outcomes of Cancer Patients in Wuhan, China. 2020:2020.03.21.20037127.

[16] Zhang L, Zhu F, Xie L, Wang C, Wang J, Chen R, et al. Clinical characteristics of COVID-19-infected cancer patients: a retrospective case study in three hospitals within Wuhan, China. Annals of oncology : official journal of the European Society for Medical Oncology. 2020.

[17] Stroppa EM, Toscani I, Citterio C, Anselmi E, Zaffignani E, Codeluppi M, et al. Coronavirus disease-2019 in cancer patients. A report of the first 25 cancer patients in a western country (Italy). Future oncology (London, England). 2020.

[18] Basse C, Diakite S, Servois V, Frelaut M, Noret A, Bellesoeur A, et al. Characteristics and outcome of SARS-CoV-2 infection in cancer patients. 2020:2020.05.14.20101576.

[19] Russell B, Moss C, Papa S, Irshad S, Ross P, Spicer J, et al. Factors affecting COVID-19 outcomes in cancer patients − A first report from Guys Cancer Centre in London. 2020:2020.05.12.20094219.

[20] Rafi Kabarriti NPB, Maxim I. Maron, Wolfgang A. Tomé, Balazs Halmos, Chandan Guha, Shalom Kalnicki, Madhur K. Garg, Nitin Ohri, . Extent of prior lung irradiation and mortality in COVID-19 patients with a cancer history. Advances in radiation oncology. 2020.

[21] Deng G, Yin M, Chen X, Zeng F. Clinical determinants for fatality of 44,672 patients with COVID-19. Critical care (London, England). 2020;24:179.

[22] Ma J, Yin J, Qian Y, Wu Y. Clinical characteristics and prognosis in cancer patients with COVID-19: A single center's retrospective study. The Journal of infection. 2020.

[23] Cook G, Ashcroft AJ, Pratt G, Popat R, Ramasamy K, Kaiser M, et al. Real-world assessment of the clinical impact of symptomatic infection with severe acute respiratory syndrome coronavirus (COVID-19 disease) in patients with Multiple Myeloma receiving systemic anti-cancer therapy.n/a.

[24] Kalinsky K, Accordino MK, Hosi K, Hawley JE, Trivedi MS, Crew KD, et al. Characteristics and outcomes of patients with breast cancer diagnosed with SARS-Cov-2 infection at an academic center in New York City. Breast cancer research and treatment. 2020:1-4.
[25] Cao B, Wang Y, Wen D, Liu W, Wang J, Fan G, et al. A Trial of Lopinavir–Ritonavir in Adults Hospitalized with Severe Covid-19. 2020;382:1787-99.
[26] Fratino L, Procopio G, Di Maio M, Cinieri S, Leo S, Beretta G. Coronavirus: Older Persons With Cancer in Italy in the COVID-19 Pandemic. Frontiers in oncology. 2020;10:648.
